# The DH31/CGRP enteroendocrine peptide triggers intestinal contractions favoring the elimination of opportunistic bacteria

**DOI:** 10.1371/journal.ppat.1007279

**Published:** 2018-09-04

**Authors:** Olivia Benguettat, Rouba Jneid, Julie Soltys, Rihab Loudhaief, Alexandra Brun-Barale, Dani Osman, Armel Gallet

**Affiliations:** 1 Université Côte d'Azur, CNRS, INRA, ISA, France; 2 Faculty of Sciences III and Azm Center for Research in Biotechnology and its Applications, LBA3B, EDST, Lebanese University, Tripoli, Lebanon; Catholic University Leuven, BELGIUM

## Abstract

The digestive tract is the first organ affected by the ingestion of foodborne bacteria. While commensal bacteria become resident, opportunistic or virulent bacteria are eliminated from the gut by the local innate immune system. Here we characterize a new mechanism of defense, independent of the immune system, in *Drosophila melanogaster*. We observed strong contractions of longitudinal visceral muscle fibers for the first 2 hours following bacterial ingestion. We showed that these visceral muscle contractions are induced by immune reactive oxygen species (ROS) that accumulate in the lumen and depend on the ROS-sensing TRPA1 receptor. We then demonstrate that both ROS and TRPA1 are required in a subset of anterior enteroendocrine cells for the release of the DH31 neuropeptide which activates its receptor in the neighboring visceral muscles. The resulting contractions of the visceral muscles favors quick expulsion of the bacteria, limiting their presence in the gut. Our results unveil a precocious mechanism of defense against ingested opportunistic bacteria, whether they are Gram-positive like *Bacillus thuringiensis* or Gram-negative like *Erwinia carotovora carotovora*. Finally, we found that the human homolog of DH31, CGRP, has a conserved function in *Drosophila*.

## Author summary

The intestine is the first barrier to fight non-commensal bacteria ingested along with the food. The innate immune system is the main mean mounted by the gut lining in response to ill-causing bacteria to avoid detrimental impact. Intestinal cells produce chlorine bleach and antimicrobial peptides that destroy exogenous bacteria. Here, we identified and characterized a new mechanism of gut defense that occurs rapidly after ingestion of exogenous bacteria. We found that the enteroendocrine cells perceive the presence of chlorine bleach in the lumen thanks to a sensor. This sensor promotes a calcium flux within enteroendocrine cells that allows the release of a hormone. This hormone acts locally on the visceral muscle surrounding the intestine by provoking its strong contractions (or spasms). We show that these strong but brief visceral contractions are helping to the quick expulsion of the ingested bacteria thus limiting their potential detrimental impact on the intestine. Markedly, the bleach-sensor is well known to be involved in pain. Therefore we have deciphered in this study a biological mechanism that has so far been described only empirically by medicine, potentially explaining intestinal pain and visceral spasms upon food poisoning.

## Introduction

The intestinal mucosa is endowed with several systems of defense to fight against the bacteria that are swallowed along with food. First of all, the gut epithelium is a sealed barrier preventing aggressors from crossing the epithelial layer [1]. Secondly, the gut lining is covered by mucus in vertebrates [2] or a peritrophic membrane in arthropods, which protects it from aggression [3, 4]. Furthermore the innate immune system produces reactive oxygen species (ROS) [5] and antimicrobial peptides (AMPs) to kill bacteria [6, 7]. Finally, the gut epithelium accelerates its cellular renewal to quickly replace damaged cells [8].

Another mechanism that is poorly understood, despite long-standing empirical evidence of its role in the eviction of pathogens, is visceral contractions or spasms. Limited data are available on the physiological and cellular mechanisms governing this process. In mammals, a denervated small intestine of guinea pig displays enhanced contractility upon infection by the parasitic nematode *Trichinella spiralis*, suggesting an intrinsic signal that triggers gut contractions. This enhanced motility is necessary for efficient elimination of the worm [9]. In rodents, it has been shown that the anterior portion of the intestine shows greater contractility following inflammation, compared to the posterior section (ileum and colon) [10]. The JAK/STAT pathway has been implicated in this hypercontractility, which is triggered by many parasitic nematodes [11, 12].

Recently, studies on *Drosophila melanogaster* have shed light on the relationship between the ingestion of pathogens and increased gut motility. Once in the gut, allochthonous bacteria (the Gram-negative bacterium *Erwinia carotovora carotovora 15* –*Ecc15*) secrete uracil which would act as a bacterial growth factor [13, 14]. The presence of uracil triggers a host immune reaction leading to the DUOX-dependent production of ROS (hypochlorous acid, HOCl) which is released in the lumen [13, 15, 16]. Induced in less than 1 hour in *Drosophila*, the production of ROS is the first immune mechanism to fight ingested bacteria [16–18]. Interestingly, Du and colleagues have shown that the evolutionarily conserved Transient Receptor Potential A1 channel (TRPA1), known to be activated by a wide spectrum of chemical and mechanical stimuli [19, 20], binds HOCl and triggers increased defecation [14]. They further showed that TRPA1 is expressed in some enteroendocrine cells (EECs) located in the anterior part of the *Drosophila* adult midgut [14], which is also the domain of HOCl release [13].

Here, we used *Drosophila melanogaster* to gain insights into the signaling mechanisms underlying midgut visceral muscle contractions upon ingestion of opportunistic bacteria. We found that the Diuretic Hormone 31 (DH31), the fly homolog of the vertebrate Calcitonin Gene-Related Peptide (CGRP) [21], produced by a subpopulation of anterior EECs, promotes strong visceral contractions less than 2 hours post-ingestion of bacteria. These visceral contractions are required to rapidly clear the ingested bacteria from the gut. Our results strongly suggest that the binding of HOCl to TRPA1 induces a calcium flux in anterior DH31-positive EECs, thus promoting DH31 release. Thereafter, DH31 binds to its receptor, DH31-R, in the neighboring visceral muscle and triggers contractions. Finally, we showed that the human CGRP displays an activity similar to DH31 and induces contractions of *Drosophila* visceral muscle.

## Results

### Ingestion of opportunistic bacteria rapidly induces local visceral muscle contractions

**Fig 1:**
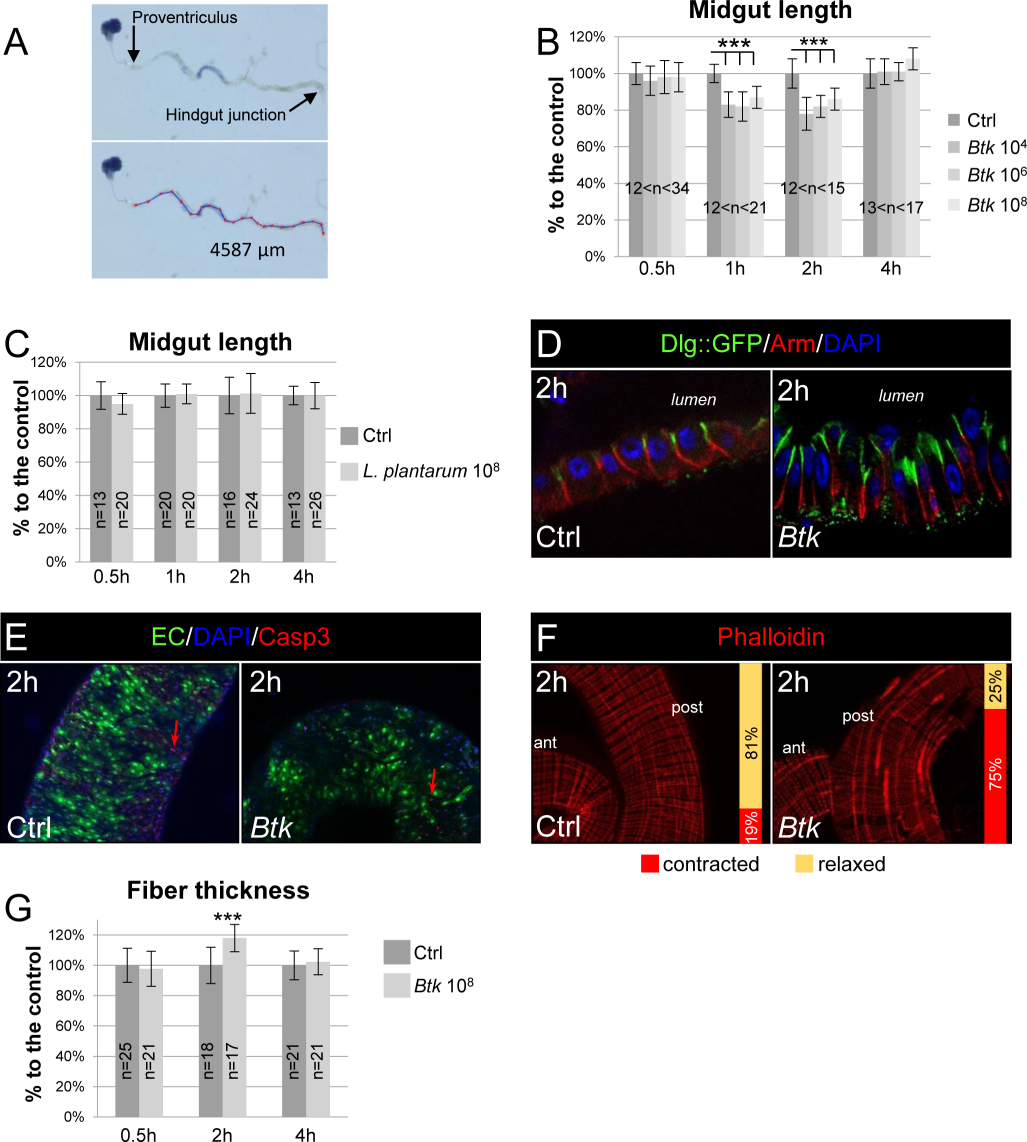
Ingestion of opportunistic bacteria rapidly induces visceral muscle spasms. (**A**) Pictures of a midgut dissected from a fly having ingested a blue food dye. The lower image shows the method to measure the length of the midgut between the proventriculus and the hindgut. (**B**) Measurement of midgut length upon intoxication by 10^4^, 10^6^ and 10^8^ CFU of *Btk*. The length is expressed in function of the control (Ctrl, 100%). Flies were fed for 30 min and the measurements were taken 30 min, 1 h, 2 h and 4 h after the initiation of feeding. (**C**) Midgut length upon intoxication by 10^8^ CFU of the commensal bacterium *L*. *plantarum*. (**D**) Immunolabelling of posterior midgut 2 h post ingestion of 5% sucrose (Ctrl) or *Btk* (10^8^ CFU/fly were provided). DAPI labels the nuclei (Blue), Arm marks the basolateral compartment (Red) and Dlg::GFP marks the apical compartment. (**E**) Caspase3 staining (Red, Casp3) of posterior midgut 2 h after feeding with 5% sucrose (Ctrl) or *Btk* (10^8^ CFU/fly were provided). Green marks the enterocytes (EC) and blue the nuclei (DAPI). Note the very few presence of apoptotic enterocytes (red arrow) in both conditions. (**F**) Visceral muscle fibers were labelled by Phalloidin 2 h after feeding with 5% sucrose (Ctrl) or *Btk* (10^8^ CFU/fly were provided). Note that only the longitudinal fibers are contracted in presence of *Btk*. Because the images are more apparent in the posterior midgut, this region was chosen to illustrate the visceral contractions in this figure and in all the following images. The vertical bars on the pictures indicate the proportion of intestines with (red) or without (yellow) contractions. (**G**) Measure of the width of the longitudinal visceral muscle fibers. Data are represented as a function of the control (Ctrl, 100%). Objective is 40X in D and 20X in E and F.

In order to determine whether the presence of opportunistic bacteria in the adult *Drosophila* midgut was able to trigger visceral muscle contractions, we first measured the length of the midgut. We have previously shown that the vegetative form of the bacterium *Bacillus thuringiensis var*. *kurstaki* (*Btk*), a Gram-positive sporulating bacillus, was cleared from the adult *Drosophila* midgut in less than 4 hours when the amount of ingested bacteria is mild [22]. These data suggested that the defense mechanisms mounted by the midgut were established quickly after *Btk* ingestion. Therefore, we carried out a dose-dependent analysis of midgut length by providing to adult flies *Btk* in quantities ranging from 10^4^ to 10^8^ CFU (Colonies Forming Units) and then measuring the length of the midgut from 30 min to 4 h post-ingestion (PI) of the bacteria (Fig 1A and 1B). To ensure accurate timing, flies were allowed contact with the contaminated medium for only 30 min (see Materials & Methods). Interestingly, we observed a significant shortening in midgut length within 1 h PI (17% at 10^4^ CFU, 18% at 10^6^ and 13% 10^8^ CFU) and this response was not dose-dependent (Fig 1B). The midgut recovered its normal length in less than 4 hours. No additional midgut shortening was observed at later time points. We also observed a similar midgut length shortening upon feeding flies with the opportunistic Gram-negative bacteria *Erwinia carotovora carotovora* 15 (*Ecc15*; S1A Fig). We also checked that this midgut shortening also occurred upon continuous feeding even though *Btk* persists for a longer time in the midgut [22]. Interestingly, we did observe both similar amplitude and timing of midgut shortening (S2B Fig). It is important to note that we did not observe any midgut shortening when flies were fed with unchallenged food (i.e. without bacteria), demonstrating that normal peristalsis does not promote significant changes in midgut length. Similarly, when flies were fed with the commensal bacteria *Lactobacillus plantarum* (*L*. *plantarum*) that persist for a longer time in the midgut (S2A Fig), we did not observe any midgut shortening (Fig 1C), highlighting the specificity of this response to opportunistic bacteria.

It has previously been shown that, upon continuous feeding with large amounts of *Ecc15*, *Drosophila* intestinal epithelial cells can delaminate as soon as 2 h PI and die by apoptosis soon after [23]. We investigated whether cell delamination and cell death could account for midgut shortening under our feeding conditions (e.g. a pulse of feeding with 10^8^ CFU of *Btk* for only 30 min). We did not observe any cell delamination (Fig 1D and S3A Fig) or increase in staining of Caspase 3 (Casp3, a marker of apoptosis; Fig 1E). However, we observed that the midgut lining was thicker with elongated cells compared to unchallenged fly midguts (Fig 1D and S3A Fig). This observation was in accordance with a narrower lumen (S2D Fig) while the midgut width was not affected by the ingestion of *Btk* (S2E Fig). Considered together, our data suggested that the shortening of the midgut could be due to strong visceral muscle contractions. We tested this hypothesis using phalloidin staining, which binds to F-Actin and therefore strongly labels the muscle fibers. We observed significant thickening of some longitudinal visceral muscle fibers 2h PI scattered along the entire length of the midgut (Fig 1F and 1G and S3B Fig) indicating local strong contractions. Indeed, we noticed that a given longitudinal fiber was not contracted all along its length and that not all the longitudinal fibers were contracted at the same time. Therefore, we can assimilate what we observed to visceral spasms. We observed similar longitudinal muscle fiber contractions upon ingestion of *Ecc15* (S1B Fig) or upon continuous feeding with *Btk* (S2C Fig). Strikingly, we never observed the thickening of circular fibers, these latter being involved in normal peristalsis following food intake. Peristalsis corresponds to a wave of contractions of circular fibers associated with a relaxation of longitudinal fibers propagating along the length of the gut, leading to overall lengthening of the intestine [24, 25]. Therefore, our results indicate that opportunistic bacteria rapidly provoke longitudinal visceral muscle contractions which do not correspond to peristalsis.

### Visceral muscle contractions help to evict opportunistic bacteria

**Fig 2:**
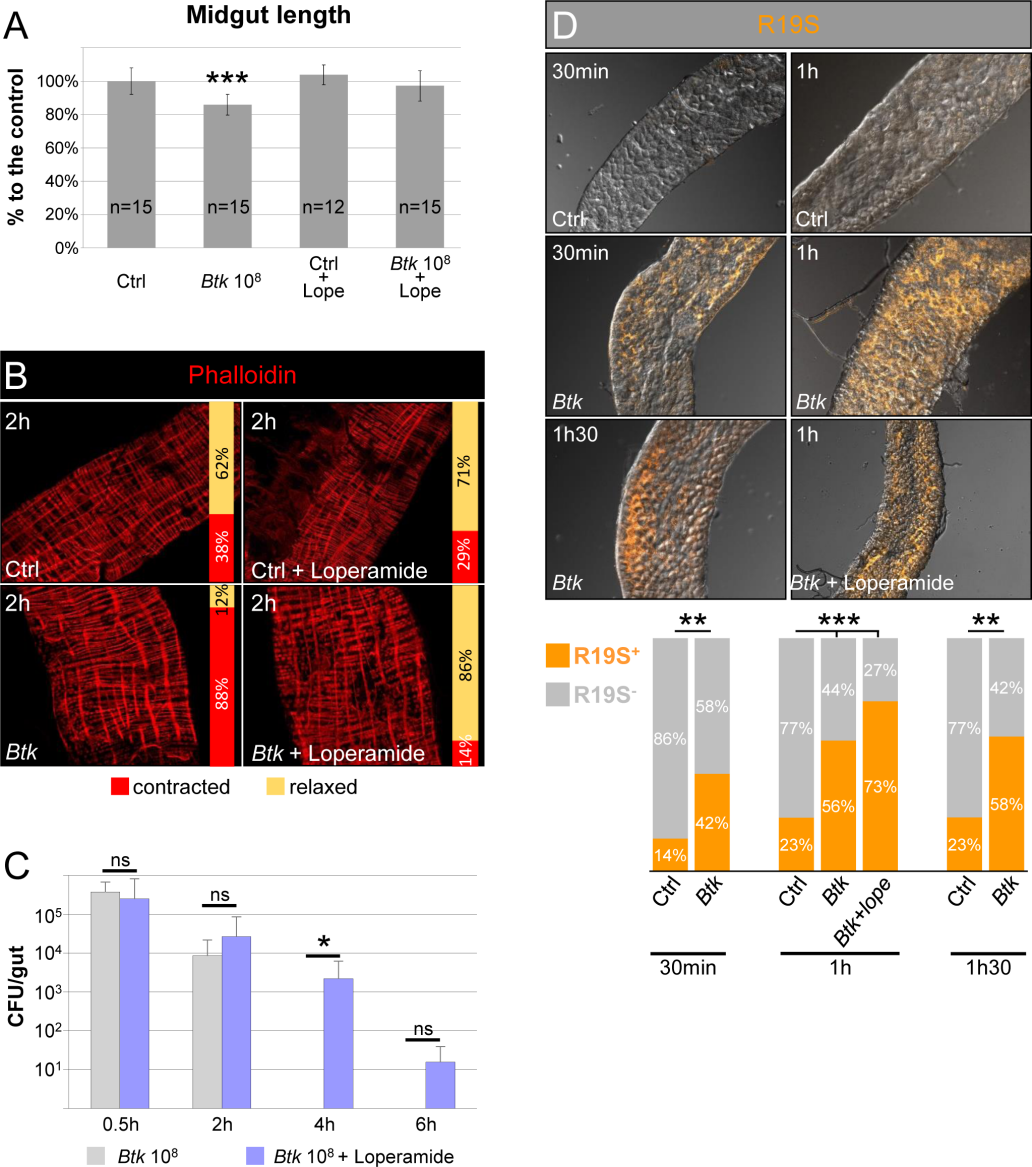
Visceral muscle spasms helps to evict opportunistic bacteria. **(A)** Measurement of the length or (**B**) phalloidin staining (red, 20X objective) of midguts from flies 2 h post-intoxication with sucrose (Ctrl), 10^8^ CFU of *Btk*, sucrose + loperamide or 10^8^ CFU of *Btk* + loperamide. (**C**) Monitoring of *Btk* persistence in the midgut of flies fed with 10^8^ CFU of *Btk* (grey bars) or 10^8^ CFU of *Btk* + loperamide (blue bars). Note that after 30 min of feeding, the same amount of *Btk* is recovered in the midgut (about 2.10^5^ CFU compared to the 10^8^ CFU/fly deposited on the medium). (**D**) Labelling of HOCl in anterior midgut by the R19-S fluorescent probe (Orange). Flies were fed with 10^8^ CFU of *Btk* or 10^8^ CFU of *Btk* + loperamide. 20X objective. The proportion of R19-S-positive (orange bars) intestines are presented in the graph below the pictures.

We sought to assess the role of these strong visceral contractions in the eviction of bacteria from the midgut. We used loperamide, a non-selective Ca^2+^ channel inhibitor that is commonly used in medicine to block spasmodic contractions of visceral muscles. As expected, the longitudinal visceral muscles were no longer contracted, whether loperamide was added alone or mixed with *Btk* (Fig 2A and 2B). We then monitored the amount of *Btk* that persisted in the midgut by counting *Btk* CFU growing from gut lysates. In absence of loperamide, *Btk* was eliminated in less than 4 h PI, whereas *Btk* persisted for at least 4 h at a dose of 2.18 10^3^ CFU in the presence of loperamide (Fig 2C). Feeding *Drosophila* with *Ecc15* led to similar observations with *Ecc15* persisting a longer time in presence of loperamide (S1C Fig). Because the persistence of the bacteria could be due to an inhibition in HOCl production (immune ROS), we verified that loperamide did not impair its production. We used the R19S probe that specifically marks HOCl in the midgut lumen [26], initially confirming that the ingestion of *Btk* induced HOCl production. As expected, HOCl was quickly detected in the anterior midgut, within 30 min PI of *Btk*, with a peak at 1 h PI (Fig 2D). In the presence of loperamide, the production of HOCl by the anterior part of the midgut was not affected (Fig 2D). Noteworthy, we do not rule out the possibility that loperamide blocks visceral spasms in *Drosophila* indirectly through unknown mechanisms. At later time point, bacteria were still eliminated (Fig 2C) demonstrating that the other mechanisms of defenses (e.g. the production of ROS and AMPs) are efficient to ultimately eliminate opportunistic bacteria. In summary, our data showed that strong longitudinal visceral muscle contractions occur between 1 and 2 h PI of opportunistic bacteria and participate in their accelerated clearance from the midgut.

### ROS signaling is required to promote visceral spasms

**Fig 3:**
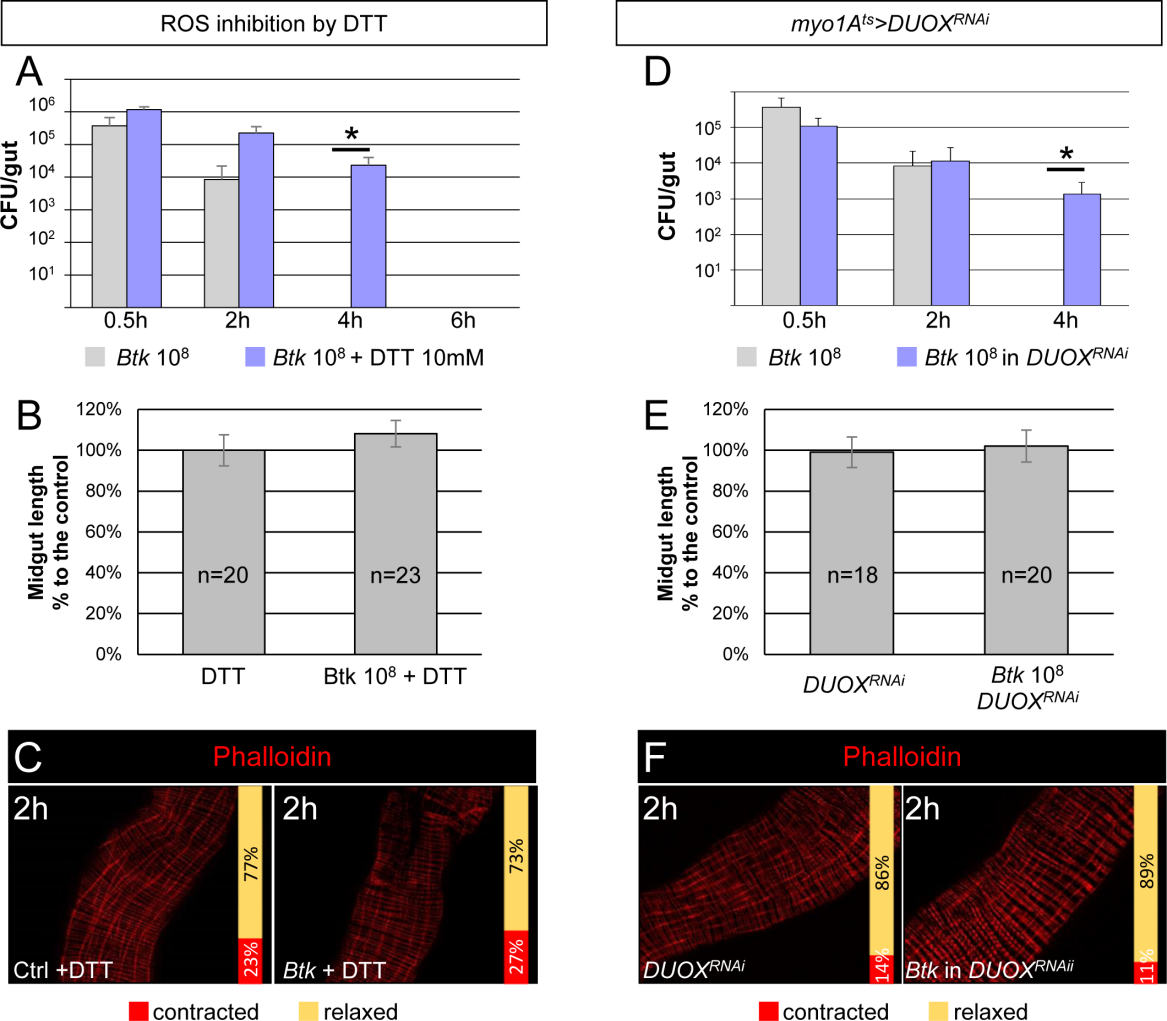
HOCl signaling is required to promote visceral spasms. (**A**) CFU counting in midguts of WT flies fed with 10^8^ CFU of *Btk* or 10^8^ CFU of *Btk* + DTT. (**B**) Measure of midgut length from flies 2 h post-intoxication with DTT (left) or 10^8^ CFU of *Btk* + DTT (right). (**C**) Phalloidin staining of posterior midgut 2 h after feeding with 5% sucrose (Ctrl) or *Btk* + DTT. 20X objective. (**D**) CFU counting in midguts of WT (grey bars) or *myo1A*^*ts*^*>DUOX*^*RNAi*^ (where *DUOX* expression was silenced in enterocytes, blue bars) flies fed with 10^8^ CFU of *Btk*. (**E**) Measure of midgut length of *myo1A*^*ts*^*>DUOX*^*RNAi*^ flies 2 h post-intoxication with sucrose (left) or 10^8^ CFU of *Btk* (right). Note that the silencing of *DUOX* in enterocytes does not affect the length of midguts compared to those from WT flies fed with 5% sucrose (Ctrl). (**F**) Phalloidin staining of posterior midgut of *myo1A*^*ts*^*>DUOX*^*RNAi*^ flies after feeding with 5% sucrose (left panel) or 10^8^ CFU of *Btk* (right panel). 20X objective.

Our next step was to identify the cellular signal that triggers the midgut spasms. As we had observed these contractions between 1 h and 2 h PI, we surmised that HOCl could be the activating cue, given that it is one of the immune components produced around 1 h PI of allochthonous bacteria [13] (Fig 2D). To determine if ROS were indeed implicated in visceral muscle contractions, we complemented fly food with the ROS-quenching dithiothreitol (DTT, 10 mM) (S4A Fig) and monitored: 1) the amount of *Btk* remaining in the midgut, 2) the midgut length and 3) the visceral muscle contractions (Fig 3A–3C). As expected, by blocking ROS activity, bacteria persisted for a longer time in the gut but, as with loperamide, were ultimately eliminated at a later time point (Fig 3A) [27]. Furthermore, we also observed an absence of midgut shortening (Fig 3B) and an inhibition of visceral muscle contractions (Fig 3C). We proceeded to confirm these results by silencing the intestinal Dual Oxidase (DUOX) enzyme which is usually expressed in enterocytes and responsible for the production of immune HOCl [5]. We specifically down-regulated *DUOX* expression in enterocytes using the *Drosophila* TARGET system [28] with the *Myo1A-Gal4*^*ts*^ promoter (see Material and methods) to drive the expression of the *DUOX*^*RNAi*^. We first confirmed that no HOCl was produced upon feeding with *Btk* (S4B Fig). As expected, we did observe an increase in *Btk* persistence (Fig 3D) and both midgut shortening and visceral muscle contractions were inhibited (Fig 3E and 3F). We also noticed during the estimation of *Btk* CFU in the midgut that there were many small colonies of *Btk* on the Petri dishes after overnight incubation when intestinal plated bacteria came from flies fed with Loperamide (production of ROS allowed but without visceral spasms) compared to flies fed with DTT (no ROS and no spasms) (S4C Fig). This observation confirmed that the presence of ROS in the lumen harms bacteria that need more time to grow once on the Petri dishes to compensate for the damages caused by the ROS [27]. Considered together, our data showed that the production of ROS by the DUOX is responsible for visceral spasms upon ingestion of *Btk*, and that both ROS production and spasms are necessary to efficiently eliminate ingested bacteria.

### TRPA1 is involved in visceral spasms and controls DH31 release

**Fig 4:**
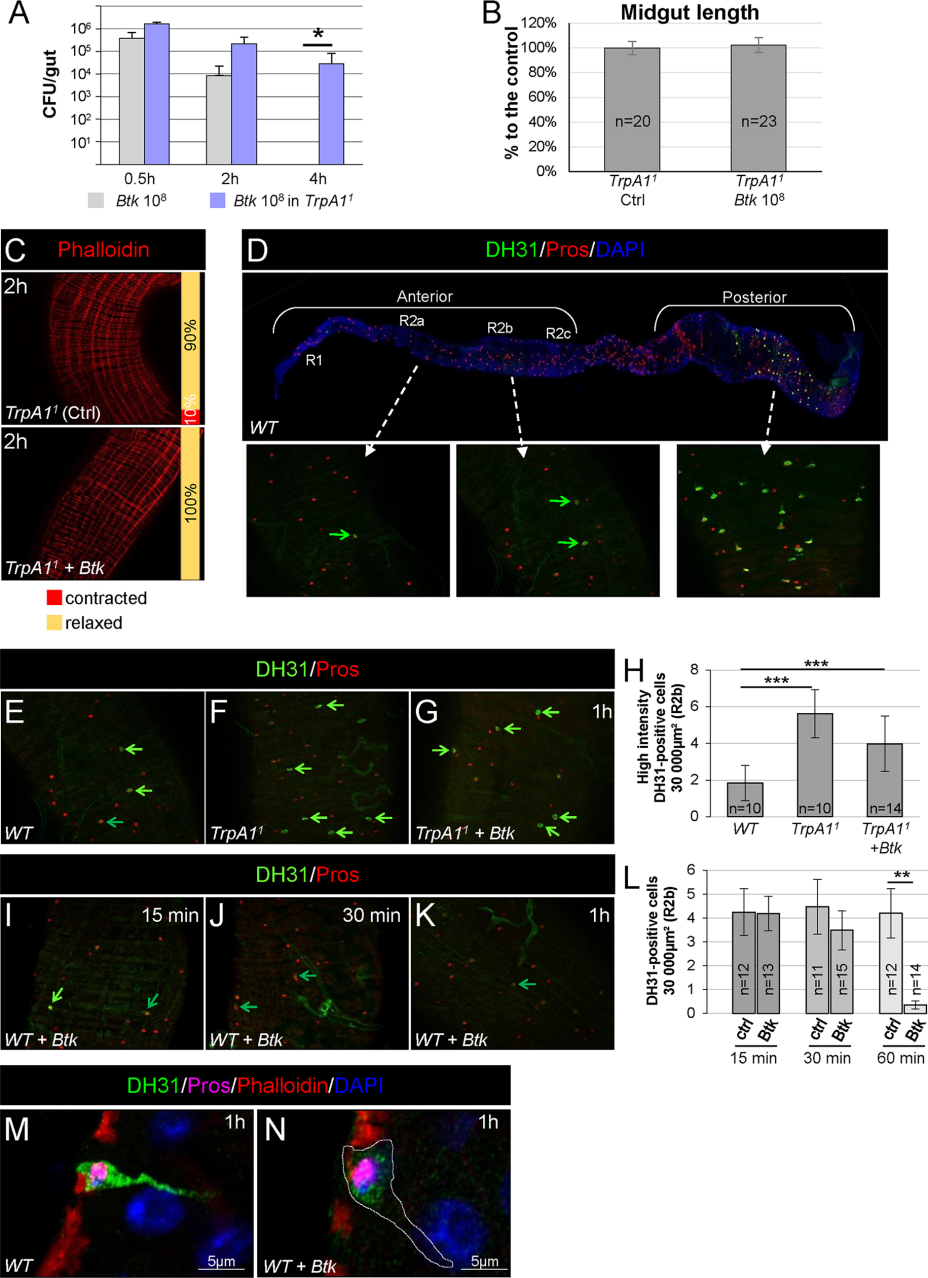
TRPA1 is involved in visceral spasms. (**A**) CFU counting in the midgut of WT (grey bars) or *TrpA1*^*1*^ homozygote (blue bars) flies fed with 10^8^ CFU of *Btk*. (**B**) Measure of midgut length of *TrpA1*^*1*^ homozygote flies 2 h post-intoxication with sucrose (Ctrl) or 10^8^ CFU of *Btk*. (**C**) Phalloidin staining of posterior midgut of *TrpA1*^*1*^ homozygote flies 2 h after feeding with 5% sucrose (Ctrl) or 10^8^ CFU of *Btk*. 20X objective. (**D**) Upper picture: reconstructed image of a WT midgut (10X objective). The midgut was stained for DH31 (Green) and Prospero (Red, Pros) that marks all the EECs. DAPI (blue) marks the nuclei. Anterior is to the left and posterior to the right. Lower insets: magnifications (40X objective) of the R2a (left), R2b (middle) and R4c (right) regions (http://flygut.epfl.ch/). (**E-G** and **I-K**) R2b anterior midgut region of WT flies (E and I-K) and *TrpA1*^*1*^ homozygote flies (F and G) fed either with sucrose (E and F) or with 10^8^ CFU of *Btk* (G and I-K). (G and I-K) midguts were dissected and fixed 15 min, 30 min and 1 h PI as indicated on the pictures. Bright green arrows point EECs with a strong DH31 labelling and dark green arrows point EECs with a lower labelling. (**H**) Counting of high intensity DH31-positive EECs in the anterior R2b domain in conditions described in (E-G). (**L**) Counting of all DH31-positive EECs in the anterior R2b domain in conditions described in (I-K). (**M** and **N**) Fast AiryScan imaging of one representative EEC in the anterior R2b midgut domain of WT flies 1h PI of sucrose (M) or 10^8^ CFU of *Btk* (N).

We investigated the signaling events that occur between ROS secretion in the lumen and the spasms of visceral muscle. It has recently been shown that the TRPA1 channel at the surface of a subset of anteriorly localized EECs was implicated in increased defecation upon ROS production [14]. Therefore we examined the role of TRPA1 in the visceral contractions of the longitudinal muscle fibers that we had previously observed. We took advantage of the homozygous viable *TrpA1*^*1*^ allele [29] to assess the TRPA1 requirement for visceral muscle spasms. Interestingly, in homozygous *TrpA1*^*1*^ midguts, *Btk* persisted for a longer time, the length was not shortened and the visceral muscle was not contracted (Fig 4A–4C).

Therefore, we wanted to identify the EEC signal that was affecting the visceral muscle. Given that EECs secrete peptides in response to many luminal inputs [30], we searched for putative enteroendocrine peptides that could regulate visceral contractions. We specifically searched for a peptide that was expressed in the EECs located in the anterior midgut because both immune ROS production [13] and HOCl-induced TRPA1 activation take place in the anterior part of the midgut [14]. We chose to focus on the DH31 peptide for several reasons. First, it has been shown that TRPA1 is implicated in the release of both DH31 in *Drosophila* [31] and CGRP in mammals [32]. Second, DH31 has been implicated in the control of visceral muscle peristalsis and motility in *Drosophila* larvae [33, 34]. Third, although DH31 is expressed in many EECs located in the posterior adult midgut, the expression of DH31 has been detected in few anterior EECs in the adult midgut [35, 36]. Fourth, two transcriptomic analyses in the *Drosophila* midgut revealed that the DH31 receptor-encoding gene *(DH31-R*) [37] is expressed in the anterior region of visceral muscle [38, 39] (http://flygut.epfl.ch; http://flygutseq.buchonlab.com).

We confirmed the expression of DH31 in anterior EECs using different sources of antibodies directed against DH31 [31, 40]. We observed a weak immuno-labelling in the anterior midgut, in contrast to the strong immuno-labelling in the posterior midgut (Fig 4D). Moreover, although we were able to detect DH31-expressing EECs in the R1 region (see http://flygut.epfl.ch/histology for the description of the midgut regions [39]), most of DH31-expressing EECs were scattered in the R2 region, with grouped cells in the R2b region (Fig 4D). We also noticed that among the R2b DH31-positve EECs there were two populations: one displaying a weak DH31 labeling (dark green arrow in Fig 4E) and one displaying a strongest labelling (bright green arrow in Fig 4E; compare Fig 4H to 4L).

We then considered whether TRPA1 might control DH31 release in response to ROS binding. Interestingly, we did observe more anterior EECs accumulating DH31 in *TrpA1*^*1*^ mutant intestines (Fig 4E, 4F and 4H). We did not observe any change in DH31 labelling in the posterior midgut (S5A–S5D Fig). Feeding *TrpA1*^*1*^ homozygous flies with *Btk* did not affect the accumulation of DH31 in anterior EECs (Fig 4G and 4H). In contrast, in a WT context, feeding flies with *Btk* or *Ecc15* promoted DH31 clearance from EECs, as DH31 became barely detectable in the anterior part of the midgut 1h PI (Fig 4I–4L and S1D and S1E Fig). Accurate observation of anterior DH31-positive EECs revealed that in absence of a stimulus (e.g. bacteria), EECs were fulfilled of DH31-containing vesicles (Fig 4M). Interestingly, we observed that the basal side of EECs were tightly intermingled with the underlying visceral muscle (Fig 4M) suggesting that DH31 could signal to the visceral muscle in a juxtacrine manner. Upon feeding with *Btk*, EECs were emptied of DH31-containing vesicles (Fig 4N). Unfortunately, we were unable to detect DH31 in neighboring cells or tissues. We assume that we reached the limit of the sensitivity of the anti-DH31 antibodies. Thus, altogether our data suggest that TRPA1 controls DH31 released from anterior EECs upon opportunistic bacterial ingestion.

### DH31 peptide is required to promote visceral spasms

**Fig 5:**
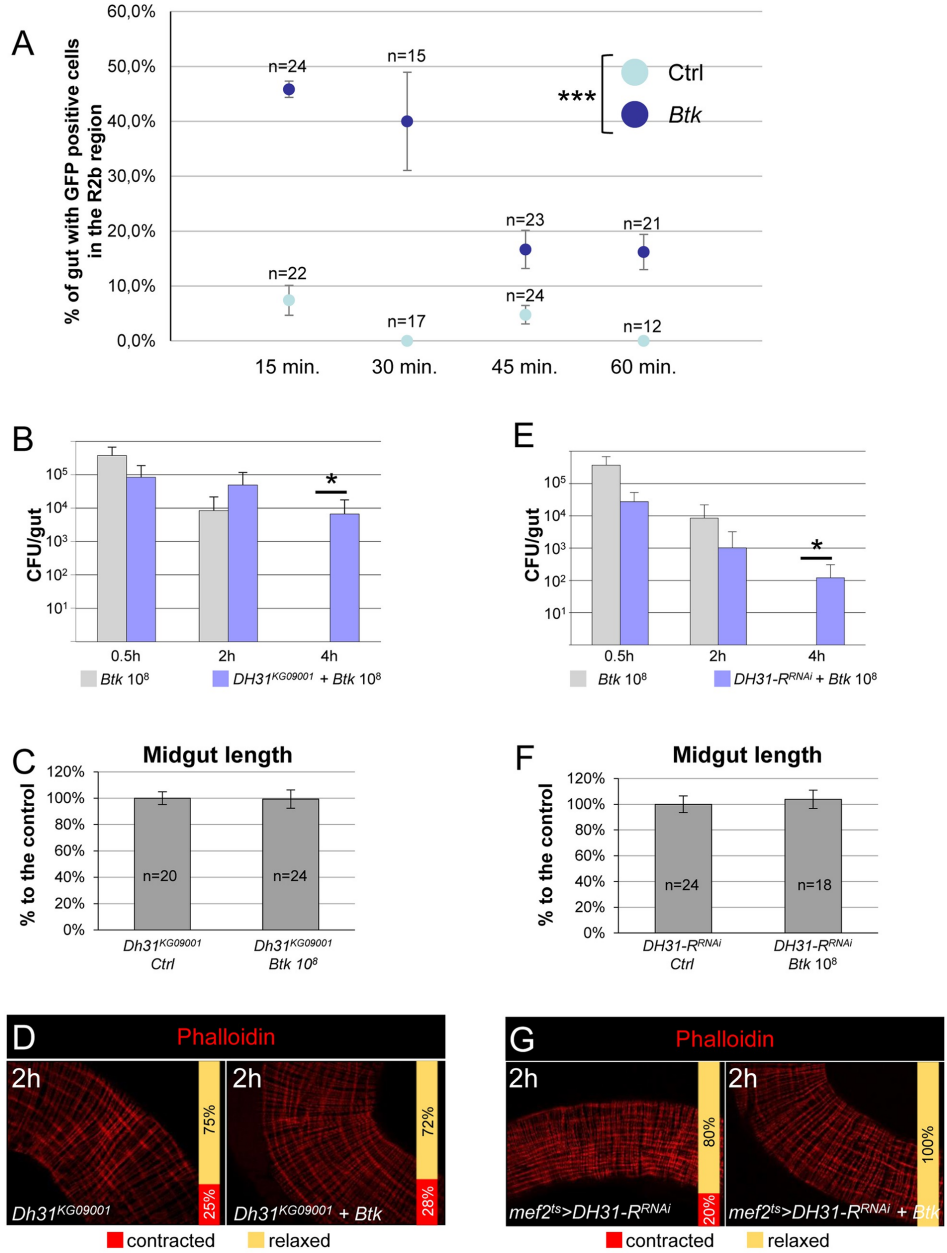
DH31 peptide is required to promote visceral contractions. (**A**) *DH31*^*ts*^*>GCaMP6s* midguts were scored for GFP-positive EECs in the R2b region in control flies (light blue) or in flies fed with 10^8^ CFU of *Btk* (dark blue). Error bars correspond to the SEM. (**B** and **E**) CFU counting in the midguts of WT (grey bars) or *DH31*^*KG09001*^ homozygote flies (B, blue bars) or *mef2*^*ts*^*>DH31-R*^*RNAi*^ flies (E, blue bars) fed with 10^8^ CFU of *Btk*. (**C** and **F**) Measure of midgut length of *DH31*^*KG09001*^ homozygote flies (C) or *mef2*^*ts*^*>DH31-R*^*RNAi*^ flies (F) 2 h post-intoxication with sucrose (Ctrl) or 10^8^ CFU of *Btk*. (**D** and **G**) Phalloidin staining of posterior midgut of *DH31*^*KG09001*^ homozygote flies (D) and *mef2*^*ts*^*>DH31-R*^*RNAi*^ flies (G) 2 h after feeding with 5% sucrose (left) or 10^8^ CFU of *Btk* (right). 20X objective.

After activation, TRPA1 mediates a Ca^2+^ flux in EECs resulting in the release of neuro- and entero- peptides [20, 30]. To show that DH31-expressing EECs were indeed responding to the presence of bacteria, we monitored their activity by imaging calcium release. We used the *DH31-Gal4*^*ts*^ transgenic line to specifically drive the expression of the GCaMP6s calcium sensor [41, 42] in DH31-expressing EECs. We first established the expression pattern of *DH31-Gal4*^*ts*^ with GFP. GFP-positive EECs were mainly present in the R2b region of the midgut but we found in half of the midguts observed an EEC positive for DH31 immunolabelling that did not express the GFP (S6A and S6A' Fig), meaning that *DH31-Gal4*^*ts*^ did not perfectly mimic DH31 endogenous expression in the anterior midgut. In the posterior midgut, *DH31-Gal4*^*ts*^ perfectly reproduced DH31 endogenous expression (S6B and S6B' Fig). Next, we monitored Ca^2+^ release in *DH31-Gal4*^*ts*^ *UAS-GCaMP6s* flies between 15 and 60 min post *Btk* ingestion by counting GFP-positive EECs in the R2b region. We assumed that the downstream Ca^2+^ release that induces DH31 secretion, should occur before the onset of visceral spasms, i.e. less than 1 h after ingestion. In control flies fed with 5% sucrose, we only detected anterior GFP-positive EECs in 2/22 midgut samples at 15 min and 1/24 midgut samples at 45 min. We never detected anterior GFP-positive EECs at 30 min and 60 min (Fig 5A). Furthermore, posterior DH31-expressing EECs always displayed Ca^2+^ release (S7A Fig). On the contrary, upon ingestion of *Btk*, we frequently detected anterior GFP-positive EECs, but no obvious change was observed in the posterior compartment in comparison to control flies (Fig 5A and S7A Fig). Interestingly, the Ca^2+^ peak was rapidly induced post-*Btk* ingestion. Anterior GFP-positive DH31-expressing EECs were readily detected 15 min PI and the Ca^2+^ release progressively ceased after 30min (Fig 5A). Thus, our data suggest that anterior DH31-positive EECs are sensitive to the presence of opportunistic bacteria and trigger an intercellular release of Ca^2+^. On the contrary, posterior DH31-positive EECs do not seem to modify their activity in the presence of opportunistic bacteria.

To demonstrate that the release of DH31 was involved in strong visceral muscle contractions and bacteria eviction, we used homozygote-viable adult flies for the strong hypomorphic mutant allele *Dh31*^*KG09001*^ [31, 33]. Interestingly in *Dh31*^*KG09001*^ homozygote adults, we observed a longer persistence of *Btk* in the gut (Fig 5B), which was associated with the disappearance of both midgut shortening and visceral spasms (Fig 5C and 5D). We confirmed these results by silencing *DH31* expression in all EECs using the *voilà-Gal4*^*ts*^ driver [43]. We first verified that *DH31*^*RNAi*^ effectively down regulated DH31 production by quantifying both the *DH31* mRNA level by RT-qPCR and the DH31 protein level using anti-DH31 antibodies (S7B and S7C Fig). As expected, down regulation of DH31 in EECs impaired bacterial eviction and prevented midgut shortening and visceral spasms (S7D–S7F Fig).

In addition, we down regulated the expression of the DH31 receptor (DH31-R) in visceral muscle using the specific *mef2-Gal4*^*ts*^ promoter to drive *DH31-R*^*RNAi*^ in the visceral muscle. Although the silencing was not total (S7B Fig; 64%), this was sufficient to impair *Btk* eviction, midgut shortening and muscle spasms (Fig 5E–5G). Therefore, our results show that the enteroendocrine peptide DH31 expressed in anterior EECs is involved in the transmission of the HOCl-dependent signal from the lumen to the visceral muscle.

### DH31 peptide is sufficient to promote strong visceral contractions

**Fig 6:**
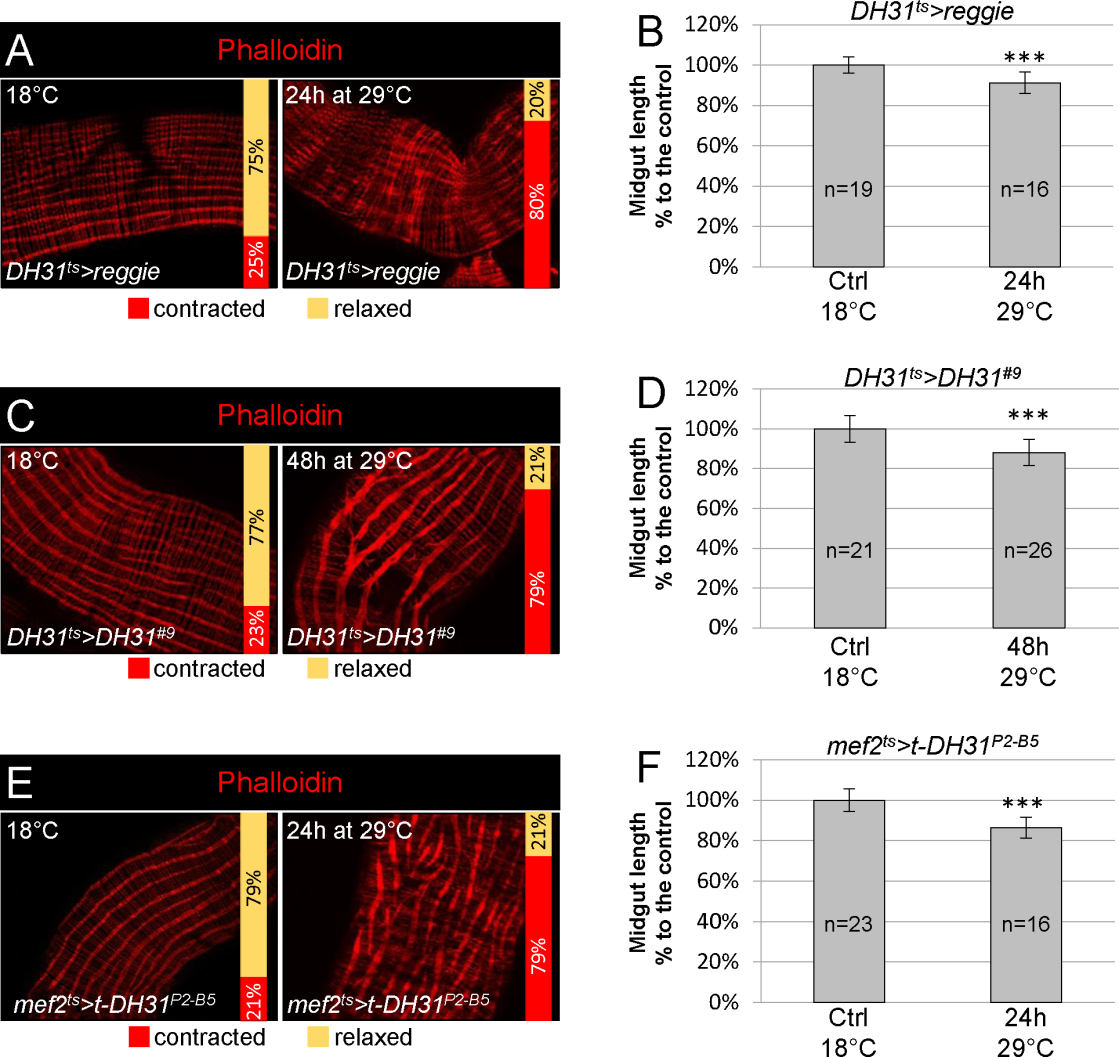
DH31 peptide is sufficient to promote visceral contractions. (**A**) Phalloidin labelling of posterior midgut of *DH31*^*ts*^*>reggie* flies maintained at 18°C (control, left) or raised for 24 h at 29°C (right). 20X objective. (**B**) Measurement of midgut length in conditions described in (A). (**C**) *DH31*^*ts*^*>DH31*^*#9*^ posterior midguts maintained at 18°C for control (left) or shifted for 48 h at 29°C (right) and stained with phalloidin. 20X objective. Overexpression of DH31 in all EECs induced strong visceral contractions. (**D**) Measurement of midgut length in the conditions described in (C). (**E**) *mef2*^*ts*^*>t-DH31*^*P2-B9*^ posterior midguts maintained at 18°C for control (left) or shifted for 24H at 29°C (right) and stained with phalloidin. 20X objective. Ectopic expression of the membrane-tethered form of DH31 in the visceral muscle induced autonomous visceral contractions. (**F**) Measurement of midgut length in the conditions described in (E).

To confirm that DH31 was sufficient to promote strong visceral contractions, we forced DH31-expressing EECs to secrete their contents by over expressing Reggie/Flotillin, a major protein component of membrane microdomains that has been shown to promote morphogen secretion [44]. We specifically expressed Reggie in DH31-expressing EECs using the *DH31-Gal4*^*ts*^ driver [36, 45]. We observed shortening of the midgut length and strong visceral contractions scattered along the entire midgut (Fig 6A and 6B), confirming that at least one factor secreted by DH31-expressing EECs was involved. We were unable to promote any strong visceral contractions by expressing Reggie in all EECs using the *voila-Gal4*^*ts*^ driver, despite testing numerous conditions. This result suggests that one or several factors secreted by different EECs could be involved in the inhibition of strong visceral contractions to counteract DH31 activity.

Then, we overexpressed DH31 in DH31-expressing EECs. We noticed that 48 h of induction at 29°C was required before we could detect a substantial increase in DH31 immuno-labelling in anterior EECs (S6C–S6D' Fig). Under these conditions, we observed strong visceral contractions and a strong midgut shortening (Fig 6C and 6D). To further demonstrate that DH31 acts on its receptor at the level of the visceral mesoderm, we expressed a membrane anchored form of DH31 (t-DH31) in the visceral mesoderm using the *mef2-Gal4*^*ts*^ driver; t-DH31 has been shown to autonomously activate its receptor DH31-R [31, 46]. Again, we observed both strong visceral contractions and a midgut shortening (Fig 6E and 6F). Finally, we wondered whether loperamide could block visceral spasms through an inhibition of DH31 secretion. To investigate this possibility, we looked at DH31 accumulation in anterior EECs 1 h PI in midguts of flies fed with both loperamide and *Btk*. We did not observe any DH31 accumulation. Instead, we obtained a clearance of DH31 from anterior EECs similar to what we got by feeding flies with *Btk* alone (Fig 4K and 4L and S5E and S5F Fig). Altogether our data demonstrate that EECs-secreted DH31 is sufficient to trigger strong visceral contractions by directly acting on the longitudinal muscles through its DH31-R receptor.

### The human DH31 homologue, CGRP, promotes strong visceral contractions

**Fig 7:**
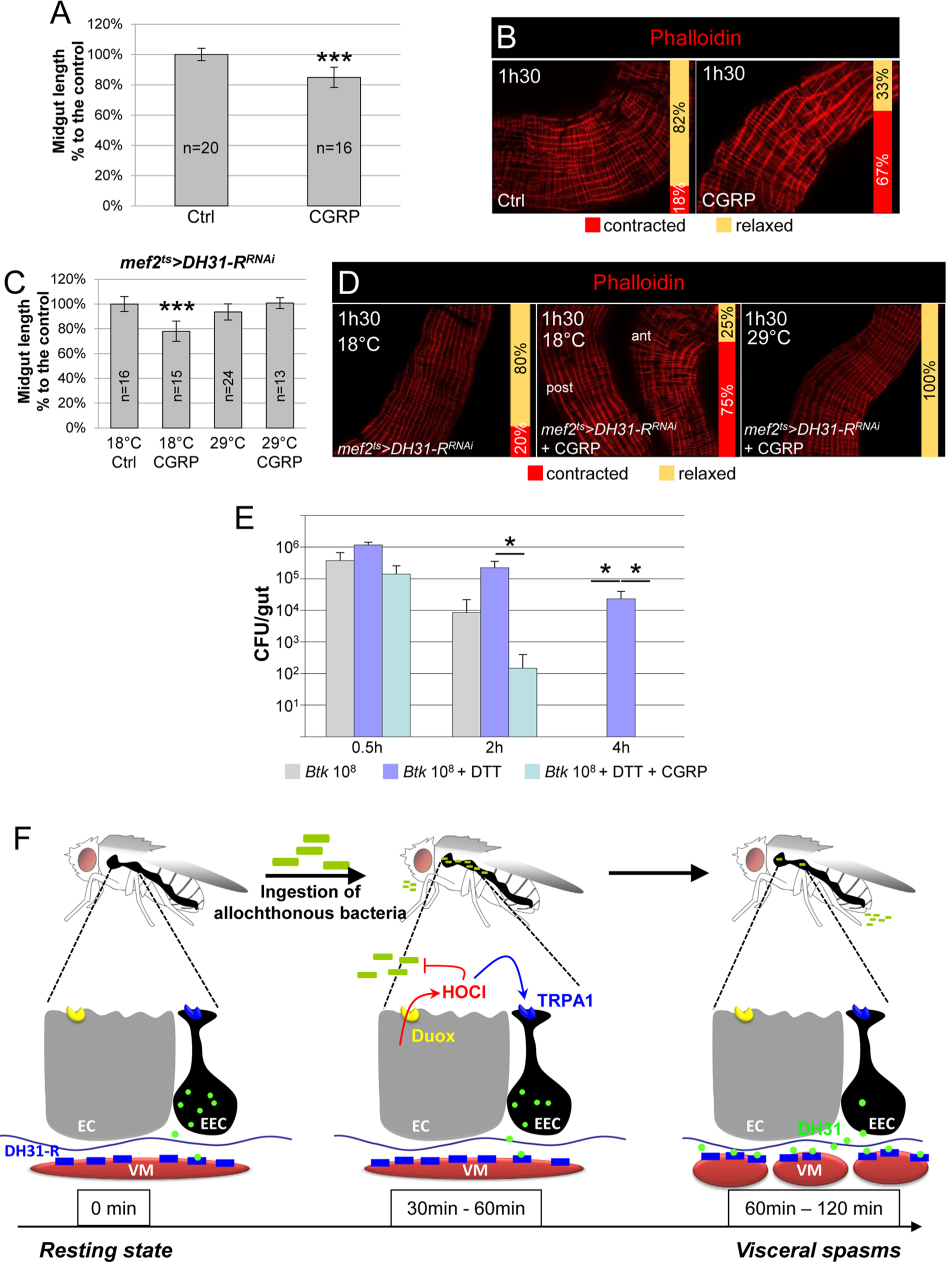
The human DH31 homolog CGRP promotes visceral contractions. (**A**) Midgut length of WT flies fed continuously either with sucrose (Ctrl) or with CGRP. (**B**) Phalloidin labelling of posterior midgut of WT flies fed continuously either with sucrose (Ctrl) or with CGRP. In (A and B) flies were dissected 1h30 after the onset of feeding. (**C**) Midgut length of *mef2*^*ts*^*>DH31-R*^*RNAi*^ flies raised at 18°C (the two left bars, Gal80^ts^ on/RNAi off) or shifted for 7 days at 29°C (the two right bars, Gal80^ts^ off/RNAi on) and fed with sucrose (first and third bars) only or with CGRP for 1h30 (second and fourth bars). The length is expressed in % to the control (Ctrl) (**D**) Phalloidin labelling of *mef2*^*ts*^*>DH31-R*^*RNAi*^ midguts from flies fed with sucrose (left) or CGRP (middle and right) and maintained at 18°C (left and middle) or shifted for 7 days at 29°C (right). (**E**) CFU counting in the midgut of WT flies fed with 10^8^ CFU of *Btk* (grey bars) or 10^8^ CFU of *Btk* + DTT (blue bars) or 10^8^ CFU of *Btk* + DTT + CGRP (turquoise bars). Note that the ingestion of CGRP can rescue the absence bacterial eviction caused by the inhibition of ROS production. (**F**) Model summarizing the sequence of events following the ingestion of opportunistic bacteria. Left: In unchallenged flies, only a little DH31 is secreted by EECs, probably involved in peristalsis. Middle: Upon ingestion of opportunistic bacteria, enterocytes (EC) release immune ROS (HOCl) in a DUOX-dependent manner to damage the bacteria. EECs sense the presence of luminal HOCl thanks to the TRPA1 receptor. Right: large amounts of DH31 are secreted by EECs. DH31 reaches the visceral muscle where it binds its receptor DH-31R, promoting spasms of the visceral muscles (VM) to expel bacteria.

While DH31 and CGRP have only weak amino-acid sequence homology [21], DH31-R and its mammalian orthologue, CALCRL [47–49], display about 34% identity and 48% similarity (S8A and S8B Fig). Therefore, we wondered whether CGRP, the mammalian DH31 homolog, might also have an effect on visceral muscle contractions. Based on previous findings in mammals [50], we fed flies with 40 μg/ml of the human αCGRP peptide. Interestingly, we observed gut shortening (Fig 7A) and strong visceral muscle contractions (Fig 7B) in a range comparable to what we observed upon ingestion of opportunistic bacteria (Fig 1) and upon overexpression of DH31 (Fig 6). To confirm that CGRP induced strong visceral contractions through binding to DH31-R, we fed *mef2*^*ts*^*>DH31-R*^*RNAi*^ knock-down flies with CGRP. In this case, the αCGRP peptide was unable to promote midgut shortening and visceral contractions when the DH31-R was downregulated in visceral muscle (Fig 7C and 7D). Finally, to demonstrate that CGRP-induced spasms could accelerate the eviction of ingested opportunistic bacteria we designed an epistasis test by blocking ROS production with DTT and feeding flies with CGRP in presence of *Btk*. As expected, the ingestion of CGRP overcame ROS inhibition and promoted an accelerated bacterial eviction (Fig 7E). Altogether, our data demonstrate that the physiological function of DH31/CGRP is conserved across orders.

## Discussion

In this study, we identified a new physiological circuit that is implicated in the local gut defense and participates in the elimination of ingested allochthonous bacteria (Fig 7F). We demonstrated that the production of immune ROS in response to the presence of allochthonous bacteria in the gut lumen leads to rapid and strong visceral contractions scattered along the midgut, which are involved in the elimination of the ingested bacteria. Most importantly, this quick and efficient response is enabled by a subset of anterior EECs that transmit the signal from the lumen to the visceral mesoderm by releasing DH31, a neuropeptide that presents a conserved function with CGRP in mammals (Fig 7F). This work reinforces the importance of EECs in the defense mechanisms of the gut, beyond the recent discovery of their involvement in innate immune response [38, 45, 51, 52] and in addition to their well-known functions in regulating feeding behavior and digestive physiology [30].

The control of DH31/CGRP secretion appears to be conserved not only in different tissues, but also across the animal kingdom. Indeed, in *Drosophila* brain, DH31 secretion is controlled by TRPA1 to promote awakening [31], and in mammals CGRP release is induced by TRPA1, for example, to control blood flow and blood pressure, and induces migraines [32, 49]. Moreover, TRPA1 belongs to the large family of pain-associated TRP receptors that act in sensitive neurons through the secretion of neuropeptides, including CGRP [19, 53]. In our study, we found that TRPA1 activation by immune ROS in a subset of anterior EECs is necessary to induce DH31 release. Surprisingly, while both immune ROS production and TRPA1 expression specifically occur in the anterior midgut (TRPA1 expression being absent from the posterior EECs [14]), the visceral spasms encompasses the whole midgut. Posterior DH31-positive EECs could contribute to the generation or propagation of spasms, although *DH31-R* is only weakly expressed in the posterior visceral mesoderm (http://flygutseq.buchonlab.com/). However, we did not observed any change in DH31 labelling in the posterior midgut upon ingestion of opportunistic bacteria. Similarly, Ca^2+^ release was differentially activated in the presence of opportunistic bacteria along the anterior-posterior axis, and was only fluctuating in anterior DH31-positive EECs. A source of DH31 from the central nervous system (CNS) is also unlikely since the *Drosophila* adult midgut is only innervated at its two extremities [39, 54]. Another possibility would be that the release of DH31 by anterior EECs (or the CNS) could enter the hemolymph and induce posterior midgut visceral contractions. Indeed, in mammals, enteroendocrine peptides can act at a very long distance after penetration in the bloodstream, and consequent activity in many organs, including the brain [55]. However, here again, the low level of *DH31-R* expression in the posterior visceral muscle does not favor this hypothesis. Thus, the simplest explanation would be that binding of DH31 to its receptor in the anterior part of the midgut triggers a myogenic propagation of the signal (as seen with peristalsis in mammals [56]), generating spasms along the midgut.

The increased gut motility in response to the presence of pathogens appears to be a conserved response. Indeed, it has been shown in rodents that, upon ingestion of the parasitic nematode *Trichinella spiralis*, the main mode of elimination is an enhanced propulsive activity of the intestine. Moreover, this physiological response is organ-autonomous since *ex-vivo* denervated intestine still responds to the presence *T*. *spiralis* [9, 57, 58]. This increased gut propulsive activity is linked to the thickening of both longitudinal and circular smooth muscle fibers [57, 59]. Recently, in *Drosophila*, it has also been shown that the opportunistic bacteria *Ecc15* induce accelerated defecation between 2 h and 8 h post-ingestion, although this was only observed in males [14]. Interestingly, this study also identified the requirement for both the immune ROS and the TRPA1 receptor to increase defecation. Blocking this accelerated defecation (i.e. in a *TrpA1* mutant) prolonged the persistence of the bacteria in the gut. Moreover, TRPA1 (isoform C in particular), expressed in a subset of anterior EECs was tissue-autonomously required [14]. These data, in rodents and in *Drosophila*, suggest a conserved function: increased gut motility that is autonomously controlled, as a means to evict pathogens from the gut. This increased gut motility occurs a long time after the ingestion of bacteria, is linked to accelerated gut peristalsis, and lasts from a few hours to a few days [14, 57]. Conversely, in our study, the visceral response occurs rapidly after bacteria ingestion, induces strong contractions of the longitudinal visceral muscle fibres and only lasts for a short period (about 1 h). Therefore, we can assume that these strong visceral contractions are the primary event evolving then towards accelerated intestinal motility/defecation until the pathogen is completely eliminated. Indeed, Du and colleagues (2016) fed flies for 2 h while we fed flies for only 30 min. When using their mode of oral intoxication (i.e. for 2 h), we also observed strong visceral contractions between 1 h and 2 h post ingestion, but we could still detect bacteria in the gut at 4 h. We believe that this likely due to the refill of the gut by newly ingested bacteria. Therefore, while the early massive visceral contractions are necessary to rapidly evict bacteria in response to a short period of intoxication, increased gut motility/defecation might be essential to expel pathogens upon prolonged ingestion or persistence in the gut. Interestingly the necessary elements are the same (i.e. ROS, EECs, TRPA1 and visceral muscles), but the switch from brief and violent visceral contractions to accelerated peristalsis may depend on the amount of DH31 that is secreted. Indeed, enteroendocrine peptides are stored in intracellular vesicles within EECs and are released as necessary [30]. We observed that upon the ingestion of bacteria, anterior EECs are almost cleared of DH31 in less than two hours, suggesting that large amounts of DH31 must initially be released to promote strong visceral contractions. Once secreted, the EECs have to refill their stock of DH31 through transcriptional activity, which takes time, therefore leaving little DH31 available for secretion. This low level of DH31 is probably insufficient to maintain strong visceral contractions, but might be sufficient to generate an accelerated, less deleterious peristalsis and consequently an increase in defecation. It is noteworthy that increased CGRP release in mammals has been associated with pain [49].

Similarly, the DH31 peptide and its CGRP orthologues in mammals have largely been implicated in gut motility/peristalsis in many different species, ranging from *Drosophila* larvae, to fish and mammals, and are always expressed in EECs [33, 34, 60–64]. Interestingly, in parallel to our observations, it has been shown that CGRP promotes a transient contraction of longitudinal muscle fibers followed by a longer phase of relaxation in the guinea pig ileum. In addition, increasing the doses of CGRP enhanced contraction at the expense of relaxation [65]. Furthermore, intraperitoneal injection of CGRP in mice can induce diarrhea, which is in agreement with our *Drosophila* data [60]. Very interestingly Yoshikawa and colleagues showed that in mice that are deficient in RAMP1 (a subunit of the CGRP receptor), there is a suppression of anaphylactic diarrhea owing to a decrease in intestinal peristalsis. There is no difference in peristalsis in WT and RAMP1-deficient mice under normal conditions [62], suggesting that CGRP is dispensable for normal peristalsis. This observation is supported by data in *Drosophila* where the *DH31*^*KG09001*^ mutant (as used in this study) is homozygote viable [31, 33]. Therefore, all these data strongly suggest that the main function of the controlled release of DH31/CGRP from EECs is to induce increased gut contractility under pathogenic conditions, in order to evict the source of the trouble. This increased gut contractility has two phases: the first phase depends on high amounts of DH31/CGRP to trigger visceral spasms, followed by accelerated peristalsis that requires lower amounts of DH31/CGRP. Our results pave the way for a functional study of the very first molecular stages of food poisoning in vertebrates.

Finally, medical diagnosis empirically links intestinal pain to visceral spasms upon food poisoning and/or diarrhea. Consequently, most of time medical professionals prescribe drugs that inhibit the visceral spasms, to treat both diarrhea and pain. However, this practice should perhaps be reconsidered since our work suggests that blocking visceral spasms probably slows down the elimination of the pathogens that are responsible for the discomfort. Therefore, our work sheds light on, and deciphers, a physiological mechanism that will be helpful to design novel drugs and adapt medical practices to treat the visceral pain and diarrhea associated with bacteria.

## Materials and methods

### Fly strains

We used the following stocks: WT canton S (Bloomington #64349); *w; DH31-Gal4* (Bloomington #46389; gift from Jae Young Kwon); *w; tub-Gal80*^*ts*^*; TM2/TM6b* (Bloomington #7019); *w; myo1A-Gal4; tubGal80*^*ts*^ *UAS-GFP* (gift from Nicolas Tapon); *y w; tubGal80*^*ts*^*; voilà-Gal4/TM6b* (gift from Marcos Vidal, [43]); *UAS-dicer2*, *w; tub-Gal80*^*ts*^*; mef2-Gal4* (Bloomington #25756); *y v; UAS-DUOX*^*RNAi*^ (Bloomington #38907 corresponding to the data presented in this article); *y v; UAS-DH31*^*RNAi*^*/TM3* (Bloomington #41957); *y v; UAS-DH31-R*^*RNAi*^ (Bloomington #259257); *w; TrpA1*^*1*^ (Bloomington #26504); *y; DH31*^*KG09001*^ (Bloomington #16474); UAS-DH31 and UAS-t-DH31 lines were gift from Fukima Hamada and were created by Paul Taghert and Mike Nitabach respectively [46, 66]; *w; 20XUAS-GCaMP6s* (Bloomington #42746, gift from N. Arquier and L. Kurz); *w; UAS-reggie-1* was obtained from Konrad Basler [44].

### Bacterial strains

The *Btk* strain (identified under the code 4D22) was provided by the Bacillus Genetic Stock Center (www.gbsc.org) and described by [67]. *Ecc15* was provided by Bruno Lemaitre’s laboratory (Ecole Polytechnique Fédérale, Lausanne, Switzerland). *L*. *plantarum* was kindly provided by Bernard Charroux (IDBM, Marseille, France). *Btk* and *Ecc15* were grown as described in Loudhaief et al. (2017). To concentrate bacteria and reach the requested OD at 600 nm, 300 mL overnight cultures were centrifuged for 10 min at 2500 rcf. The pellet of centrifuged bacteria was diluted in 5% sucrose in water to reach the desired concentrations.

### *Drosophila* rearing and intoxication

*Drosophila* were reared on standard medium at 25°C. For the Gal80^ts^ experiments, F1 flies were reared at 18°C and then shifted to 29°C to alleviate Gal80^ts^ repression on Gal4. For oral intoxication, after 2 h of starvation, 5- to 7-day-old females (at 25°C) were flipped onto fly medium covered with filter disks soaked in a mix of bacteria and 0.5% bromophenol blue for 30 min only. Concentrations of bacteria used: 1.10^4^ CFU/5 cm^2^/fly, 1.10^6^ CFU/5 cm^2^/fly and 1.10^8^ CFU/5 cm^2^/fly.

### RNAi experiments

For RNAi experiments newly hatched F1 females were reared at 18°C until they reached 10 days old. Then *myo1A*^*ts*^*>DUOX*^*RNAi*^ females were shifted to 29° for 4 days before bacterial feeding, *violà*^*ts*^*>DH31*^*RNAi*^ females were shifted to 29° for 4 days before bacterial feeding and *mef2*^*ts*^*>DH31-R*^*RNAi*^ females were shifted to 29° for 7 days before bacterial feeding.

### Overexpression experiments

*DH31*^*ts*^*>DH31*, *DH31*^*ts*^*>reggie*, *voilà*^*ts*^*>reggie* and *mef2*^*ts*^*>t-DH31* F1 females were reared at 18°C and 10 day-old F1 females were shifted to 29° C for 24 h or 48 h before dissection. The *UAS-DH31* #9 and #18 were used. The data presented are those from line #9. The *UAS-t-DH31* #P2-B4 and #P2-B5 were used and the data presented are those from line #P2-B5.

### Colony-forming unit (CFU) counting

Flies of different genotypes fed with *Btk*, *L*. *plantarum* or *Ecc15* were washed in 70% ethanol and PBS before dissection of guts in PBS. Guts were crushed in 200 μL of LB or MRS medium at various times after intoxication using a micropestle, and the homogenate was serially diluted in LB or MRS medium and incubated overnight at 30°C (*Btk*) or 37°C (*L*.*plantarum*) on LB (*Btk* and *Ecc15*) or MRS (*L*. *plantarum*) agar plates. Colony counting were performed the day after.

### Loperamide, DTT and CGRP feeding

Loperamide (Sigma #L4762) was resuspended in distilled water and then diluted extemporaneously in 5% sucrose. We tested two different doses: 40 μg/ml and 0.4 μg/ml. We noticed that at 40 μg/ml, food intake was reduced. Therefore, we used the lower dose (0.4 μg/ml) that did not impair food intake. For intoxication, loperamide were added to the bacterial mixture in 5% sucrose. DTT was resuspended in distilled water. We used a dose of 10 mM in 5% sucrose. After 30 min of feeding with the mixture containing bacteria and DTT, flies were flipped onto media still containing 10 mM DTT, to allow a continuous neutralization of ROS. CGRP (Sigma #C0167) was resuspended in distilled water. 3 doses were tested: 400 μg/ml, 40 μg/ml and 0.4 μg/ml. We did not observe any effect with the lower dose. The two other doses (400 μg/ml and 40 μg/ml) displayed similar results. After 2 h of starvation, flies were continuously fed with 40 μg/ml of CGRP mixed in 5% sucrose. Dissections were performed 1.5h post-ingestion. Co-feeding with DTT and CGRP: Flies were first fed for 30 min with a mixture of 5% sucrose containing 10^8^ CFU of *Btk*, 10 mM DTT and 40 μg/ml of CGRP. After 30min, flies were flipped onto media still containing 10 mM DTT and 40 μg/ml of CGRP in 5% sucrose but without *Btk*.

### Measurement of gut length

Guts were dissected and fixed with 4% formaldehyde in PBS for 15 min and immediately mounted in 80% glycerol/PBS. Guts were observed with a numeric Keyence VHX 2000 microscope. Images were analyzed using the VHX-2000 software. The intestine length is shown as a percentage relative to the control (at 100%).

### HOCl staining with R19-S

R19-S was purchased from Futurechem (FC-8001, Seoul, South Korea). For microscope scanning of the R19-S fluorescence signal, 50 μM of R19-S was added to the 5% sucrose solution (with or without bacteria) deposited on the filter disk. Flies were allowed to feed for 30 min on the mix (5% sucrose + bacteria + R19-S). Then flies were transferred to a new vial with a filter disk only soaked with R19-S in 5% sucrose. Flies were then dissected at the desired time points. The guts were fixed in 4% formaldehyde in PBS for 50 min. Guts were mounted in 80% Glycerol/PBS and immediately observed on a Zeiss Axioplan Z1 with Apotome 2 microscope.

### Dissection, immunostaining and image capture

Dissection, fixation and immunostaining were performed as described by [68]. The following antibodies were used: mouse anti-Prospero (MR1A-c, Developmental Studies Hybridoma Bank (DSHB)) at 1:200; mouse anti-Arm (N2 7A1-s, DSHB) at 1:50; rabbit anti-Caspase3 (Cell Signaling, #9661) at 1:300; rabbit anti-DH31 (gift from Jan Veenstra [40]) and Michael Nitabach [31]) at 1:500. The secondary antibodies used were anti-mouse Alexa647, anti-rabbit Alexa488, anti-rabbit Alexa546 (Invitrogen). All secondary antibodies were used at 1:1000. Phalloidin-Alexa555 (Molecular Probes, A34055) were used at 1:500 2h at room temperature or 1:2000 overnight at 4°C. Guts were mounted in Fluoroshield-DAPI medium (Sigma) and observed with a Zeiss Axioplan Z1 with Apotome 2 microscope. Pictures in Fig 4M and 4N were acquired using a Zeiss LSM 880 confocal equipped with a Fast AiryScan. Images were analyzed using ZEN (Zeiss) and Photoshop software. Image acquisition was performed at the Microscopy platform of the Institut Sophia Agrobiotech (INRA 1355-UNS-CNRS 7254-Sophia Antipolis).

### Measurement of muscle fiber thickness

After dissection of the midgut, Phalloidin labelling and image capture, three independent longitudinal fibers were measured per intestine using Zen software. Fibers were chosen in the posterior midgut and in a domain displaying contractions.

### Calcium release analysis

*20XUAS-GCaMP6s/tub-Gal80*^*ts*^, *Dh31-Gal4/+* (GCaMP6s) F1 females were reared at 18°C for 10 days after hatching. Then GCaMP6s females were shifted to 29°C for 5 days before oral intoxication. After 2h of starvation GCaMP6s females were flipped onto fly medium covered with filter disks soaked in sucrose 5% + *Btk* at 1.10^8^ CFU/5 cm^2^/fly. Control flies were deposited on a filter disk soaked in 5% sucrose. No bromophenol blue was added to avoid fluorescence attenuation. Flies were left in contact with the filter disks for 30 min, with the exception of the 15 min time point. Midguts were then quickly dissected in PBS 1X and fixed in 4% formaldehyde/PBS for 5 min (not more because a longer time of fixation induces GCaMP6s fading). The guts were rapidly mounted in 50% glycerol/PBS and immediately observed with a Zeiss Axioplan Z1 with Apotome 2. The R2b and the R4c regions were examined for green fluorescence emission.

### RT-qPCR

Total RNA was extracted from 10 midguts of *D*. *melanogaster* strains using TRIzol Reagent (Invitrogen Co.,USA) according to the manufacturer’s instructions. First strand cDNA was synthesized from total RNA (500 ng) using the qScript^TM^ cDNA SuperMix Kit (Quanta BioSciences, Inc., USA) following the manufacturer’s instructions. Quantitative real time PCR was performed using specific primers for *DH31* (*DH31*forward tcctcctcttctgcctcttg, *DH31*reverse gcacctcctccagttcgtt) and *DH31-R* (*DH31-R*forward gatggctggctttgttgg, *DH31-R*reverse cgagaccgcattccttgt) genes. The amplification efficiency of each gene was estimated by using the equation E = 10-1/slope, where the slope was derived from the plot of amplification critical time (Ct value) versus serially diluted template cDNA. The PCR master mix (20 μl) contained cDNA (5 μl) that had been diluted 10 fold, 5x HOT Pol EvaGreen qPCR Mix Plus (Euromedex, France) (5 μl) and each gene specific primer (3.6 mM). The amplification conditions were 15 min at 95°C to activate the polymerase and denature the sample followed by 40 cycles at 95°C for 15 sec, 60°C for 20 sec and 72°C for 20 sec. Quantitative PCR was performed using a continuous fluorescence detector, Aria Mx Real Time PCR system (Agilent Technologies, USA). After each quantitative PCR reaction, a melting curve was performed in order to verify that the amplicon was at the correct Tm and therefore confirming the correct length of the predicted transcript. Results were normalized to the mRNA level of two housekeeping genes, RP49 ribosomal protein *L32* gene (GenBank accession nos. NM_079843: RP49forward cgcaccaagcacttcatc, RP49reverse cactctgttgtcgatacccttg), *dp1* dodeca-satellite binding protein 1 gene (GenBank accession nos. NM_079057: *dp1*forward acgggcagaattgagaagtg, *dp1*reverse ggtacgatggaggtcgaaag), and calculated according to the delta-delta Ct method [69]. Each experiment was repeated with three independent mRNA samples (biological replicates) and each reaction was repeated three times to minimize intra-experiment variation (technical replicates).

### Frozen sections

The protocol is described in Loudhaief et al. (2017)[22].

### DH31-positive EEC counting

EECs were double labelled with Pros and DH31. Counting was performed in the R2b region (http://flygut.epfl.ch/histology). As the surface of the R2b region differs from one gut to another, we normalized the counts relative to a defined surface (e.g. 30 000 μm^2^).

### Statistical analysis

For gut length, muscle fiber thickness and DH31-positive cell counting, statistical analysis were performed using a parametric T test (for pairwise comparison) or parametric Tukey Test (for multiple pairwise comparison) using Kyplot. For CFU counting analyses, we used the non-parametric pairwise comparisons of the Wilcoxon-Mann-Whitney test. *** (P< = 0.001); ** (P< = 0.01), * (P< = 0.05). For calcium release analysis, the p value (p = 0.0003625) was calculated with the Exact Two-Sample Fisher-Pitman Permutation Test for two independent samples (control and *Btk*-treated, stratified by time) performed with the R software.

## Supporting information

S1 Fig *Ecc15* induces similar physiological responses as *Btk*. (**A**) Measure of midgut length upon intoxication by 108 CFU of *Ecc15*. The length is expressed in function of the control (Ctrl, 100%). Flies were fed for 30min and the measures were taken 1h30 after the beginning of feeding. We chose 1h30 because this correspond to the middle of the meantime of spasms. (**B**) Visceral muscle fibers were labelled by Phalloidin 1h30 after feeding with 5% sucrose (Ctrl) or *Ecc15* (108 CFU/fly were provided). The posterior midgut are shown here. (**C**) Monitoring of *Ecc15* persistence in the midgut of flies provided with 104 CFU of *Ecc15* and complemented (blue bars) or not (grey bars) with loperamide. Note that the loperamide increased the persistence of *Ecc15* (4h instead of 2h in absence of loperamide). For CFU estimation in the midgut, we chose to provide only 104 CFU of *Ecc15* to flies because we noticed that providing higher amounts of *Ecc15* increased the variability of the number of CFU recovered in the gut at any time. We attributed this variability to the food repellent impact that increasing doses of *Ecc15* have on *Drosophila* feeding ([70]). (**D**) R2b anterior midgut region of WT flies fed either with sucrose (left panels) or with 108 CFU of *Ecc15* (right panels). Midguts were dissected and fixed 30min and 1h PI as indicated on the pictures. (**E**) Counting of DH31-positive EECs in the anterior R2b domain in conditions described in (D). Control experiments were pooled together.
(TIF)

S2 Fig Ingestion of opportunistic bacteria rapidly induces huge visceral muscle spasms. (**A**) Monitoring of *L*. *plantarum* persistence in the midgut of flies fed with 108 CFU of *L*. *plantarum*. Flies were left in contact with *L*. *plantarum* for only 30min. Unlike *Btk* and *Ecc15*, *L*. *plantarum* persists at least 24h in the midgut. (**B**) Measure of midgut length upon continuous intoxication by 108 CFU of *Btk* (light grey bars) compared to the length of unchallenged control midguts (dark grey bars). (**C**) Phalloidin labelling of visceral muscle fibers of midguts from control unchallenged flies or from flies continuously fed with 108 CFU of *Btk*. (**D** and **E**) Measure of lumen (D) and midgut (E) widths 2h after intoxication by 108 CFU of *Btk*. The widths are expressed in function to the control (Ctrl, 100%). Flies were fed for 30min and the measures were taken 2h after the beginning of feeding.
(TIF)

S3 Fig Ingestion of opportunistic bacteria induces changes in epithelial cell shape. (**A**) Transversal cross-section of 3 independent posterior midguts 2h post ingestion of 5% sucrose (Ctrl) or *Btk*. DAPI labels the nuclei (Blue), Arm marks the basolateral compartment (Red) and Dlg::GFP marks the apical compartment. Objective is 40X. Note the elongated shapes of enterocytes in *Btk*-fed conditions. (**B**) Reconstructed image of midguts (10X objective) labelled with Phalloidin. 2h after ingestion of *Btk* some longitudinal muscle fibers are contracted. These contractions are scattered all along the midgut (white arrowheads in right panel). Note also that the midgut is bent in the zones of strong visceral contractions (red arrows).
(TIF)

S4 Fig HOCl signaling is required to promote visceral contractions. (**A** and **B**) Labelling of HOCl in anterior midgut by the R19S fluorescent probe (Orange) 1h after ingestion of sucrose 5% (Ctrl) or *Btk* (108 CFU were provided). *Btk* ingestion induces a production of ROS in the anterior part of the midgut (compared middle panels to left panels). Co-ingestion of DTT (A) or silencing *DUOX* expression in enterocytes (*myo1Ats>DUOXRNAI*) (B) neutralizes the production of ROS normally induced by *Btk* (right panels). Graphs on the right represent the proportion of R19S-positive midguts (orange). (**C**) Petri dishes plated with midgut lysates coming from flies co-fed with *Btk* (108 CFU) and DTT (left panel) or loperamide (middle and right panels). Note that the production ROS (loperamide condition) impairs bacterial growth as illustrated by the presence of small *Btk* colonies (red arrows).
(TIF)

S5 Fig Posterior DH31 accumulation is not affected by the ingestion of *Btk*. (**A-F**) Immunolabelling against DH31 (green) and Pros (red). DAPI (blue) marks the nuclei. (**A** and **B**) Posterior midguts of WT flies 1h post ingestion of sucrose (A) or 108 CFU of *Btk* (B). (**C** and **D**) Posterior midguts of *TrpA11*homozygote flies 1h post ingestion of sucrose (C) or 108 CFU of *Btk* (D). (**E** and **F**) Anterior midguts of WT flies 1h post ingestion of loperamide (E) or 108 CFU of *Btk* in presence of loperamide (F).
(TIF)

S6 Fig DH31 peptide is sufficient to promote visceral contractions. (**A-D'**) Anti-Pros (turquoise) and anti-DH31 (red) immunostaining in *DH31ts>GFP* (A-B) or *DH31ts>DH31* (C-D) midguts. Nuclei are marked with DAPI (blue). 40X objective. (**A-A'**) R2b region in the anterior midgut. Note that in some GFP-expressing EECs, DH31 is below the threshold of detection (green arrow). There are also few EECs where DH31 is detectable without being marked by the GFP (red arrow) suggesting that the *DH31-Gal4* driver (Bloomington stock #46389) does not perfectly recapitulate endogenous DH31 expression in the anterior midgut. (**B-B'**) R4 region in the posterior midgut. GFP expression perfectly overlaps DH31-positive EECs. (**C-C'**) Notable DH31 overexpression in the R2b region relative to the level of endogenous expression (compare C' to A'). (**D-D'**) Overexpression of DH31 in the R4 region compared with (B-B').
(TIF)

S7 Fig DH31 peptide is required to promote visceral contractions. (**A**) Ca2+ release imaging (GFP) in *DH31Gal4ts>GCaMP6s* midguts. R2b and R4c regions were captured 15 min. and 1h post ingestion of *Btk*. Ctrl corresponds to flies fed with sucrose. (**B**) RT-qPCR on *voilats>DH31RNAi* or *mef2ts>DH31-RRNAi* whole midguts compared to RT-qPCR on their RNAi bearing parents. Normalized expression of *DH31* (left) and *DH31-R* (right) are shown. (**C**) DH31 (green) and Pros (red) double immuno-labelling in WT (left) or *voilats>DH31RNAi* (right) posterior midgut. After 4 days of silencing, DH31 peptide is barely detectable in posterior EECs. Blue (DAPI) marks the nuclei. 40X objective. (**D**) CFU counting in the midgut of control (grey bars) or *voilats>DH31RNAi* flies (blue bars) fed with 108 CFU of *Btk*. (**E**) Measure of midgut length 2h post-intoxication of *voilats>DH31RNAi* flies fed or not with 108 CFU of *Btk* and compared to midgut length of control flies fed with 5% sucrose. (**F**) Phalloidin staining of posterior midgut of *voilats>DH31RNAi* flies 2h after feeding with 5% sucrose (left) or 108 CFU of *Btk* (right). 20X objective.
(TIF)

S8 Fig Sequence alignments between DH31-R and human CALRL. (**A**) The DH31-R isoform A amino acids sequence was aligned with human CALCRL amino acids sequence using needle program (*http://emboss.toulouse.inra.fr/*) with the following parameters: Matrix: EBLOSUM62; Gap penalty: 15.0; Extend penalty: 0.5. The two coding sequences present 34.5% of identity and 48.4% of similarity. (**B**) Alignment of the three DH31-R isoforms with human CALCRL (*http://multalin.toulouse.inra.fr/multalin/*). In red are shown the conserved amino acids.
(TIF)
