# Supplementary figures and images for: The DH31/CGRP enteroendocrine peptide triggers intestinal contractions favoring the elimination of opportunistic bacteria
Source: PLoS Pathog. 2018 Sep 4;14(9):e1007279. doi: 10.1371/journal.ppat.1007279 (PMC6138423; doi:10.1371/journal.ppat.1007279)

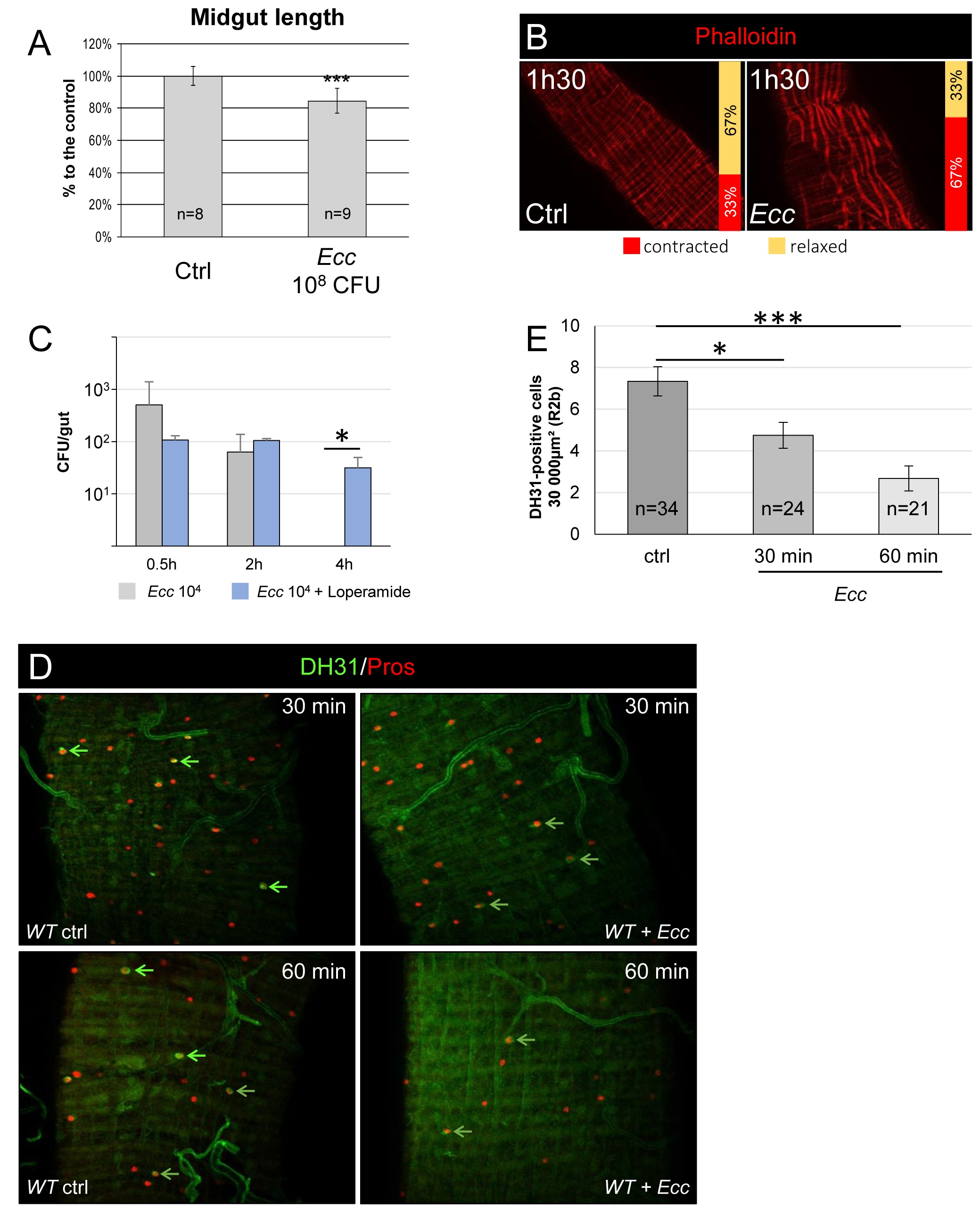

Supplement: S1 Fig — (A) Measure of midgut length upon intoxication by 108 CFU of Ecc15. The length is expressed in function of the control (Ctrl, 100%). Flies were fed for 30min and the measures were taken 1h30 after the beginning of feeding. We chose 1h30 because this correspond to the middle of the meantime of spasms. (B) Visceral muscle fibers were labelled by Phalloidin 1h30 after feeding with 5% sucrose (Ctrl) or Ecc15 (108 CFU/fly were provided). The posterior midgut are shown here. (C) Monitoring of Ecc15 persistence in the midgut of flies provided with 104 CFU of Ecc15 and complemented (blue bars) or not (grey bars) with loperamide. Note that the loperamide increased the persistence of Ecc15 (4h instead of 2h in absence of loperamide). For CFU estimation in the midgut, we chose to provide only 104 CFU of Ecc15 to flies because we noticed that providing higher amounts of Ecc15 increased the variability of the number of CFU recovered in the gut at any time. We attributed this variability to the food repellent impact that increasing doses of Ecc15 have on Drosophila feeding ([70]). (D) R2b anterior midgut region of WT flies fed either with sucrose (left panels) or with 108 CFU of Ecc15 (right panels). Midguts were dissected and fixed 30min and 1h PI as indicated on the pictures. (E) Counting of DH31-positive EECs in the anterior R2b domain in conditions described in (D). Control experiments were pooled together. (TIF) [file ppat.1007279.s001.tif]

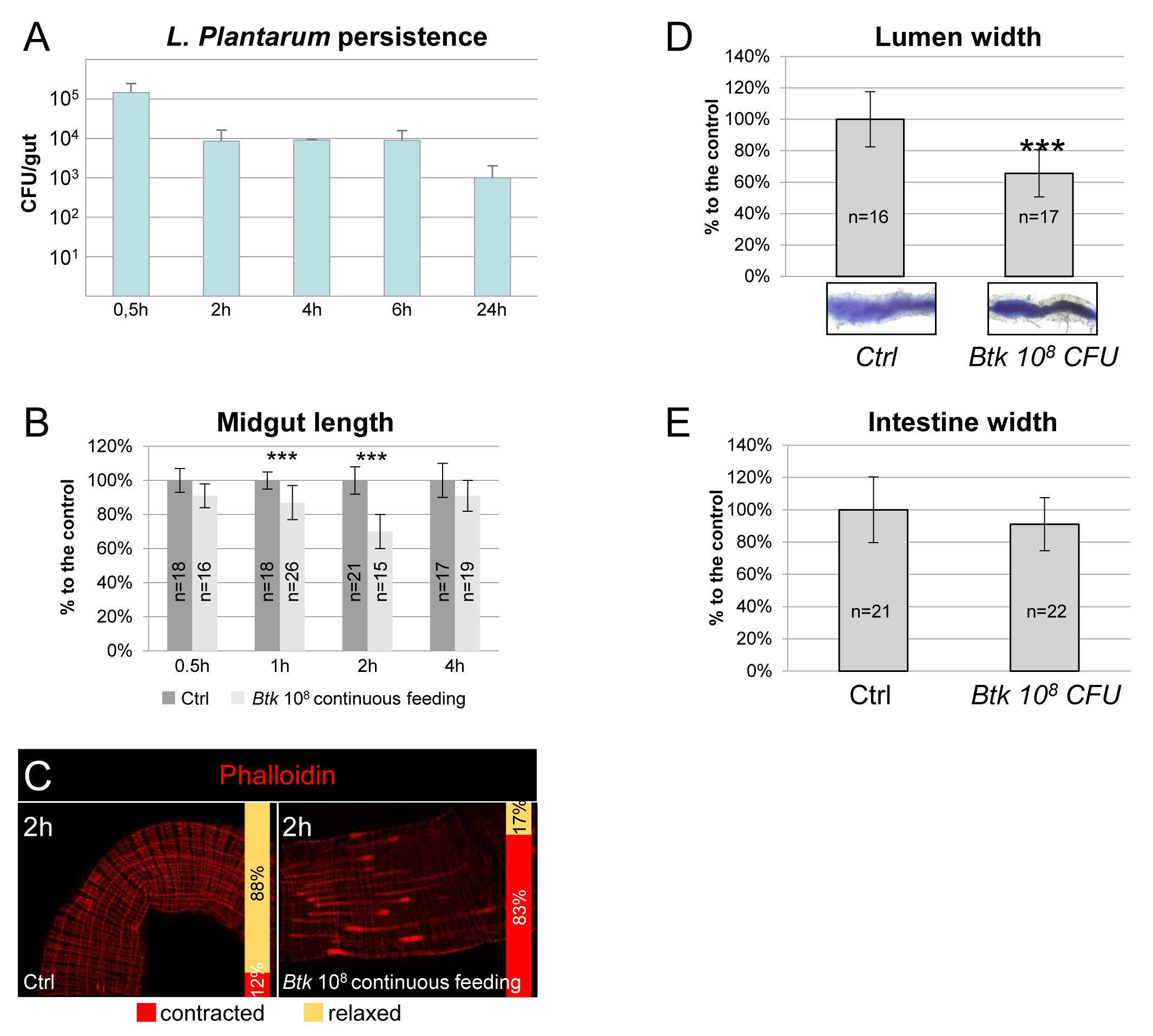

Supplement: S2 Fig — (A) Monitoring of L. plantarum persistence in the midgut of flies fed with 108 CFU of L. plantarum. Flies were left in contact with L. plantarum for only 30min. Unlike Btk and Ecc15, L. plantarum persists at least 24h in the midgut. (B) Measure of midgut length upon continuous intoxication by 108 CFU of Btk (light grey bars) compared to the length of unchallenged control midguts (dark grey bars). (C) Phalloidin labelling of visceral muscle fibers of midguts from control unchallenged flies or from flies continuously fed with 108 CFU of Btk. (D and E) Measure of lumen (D) and midgut (E) widths 2h after intoxication by 108 CFU of Btk. The widths are expressed in function to the control (Ctrl, 100%). Flies were fed for 30min and the measures were taken 2h after the beginning of feeding. (TIF) [file ppat.1007279.s002.tif]

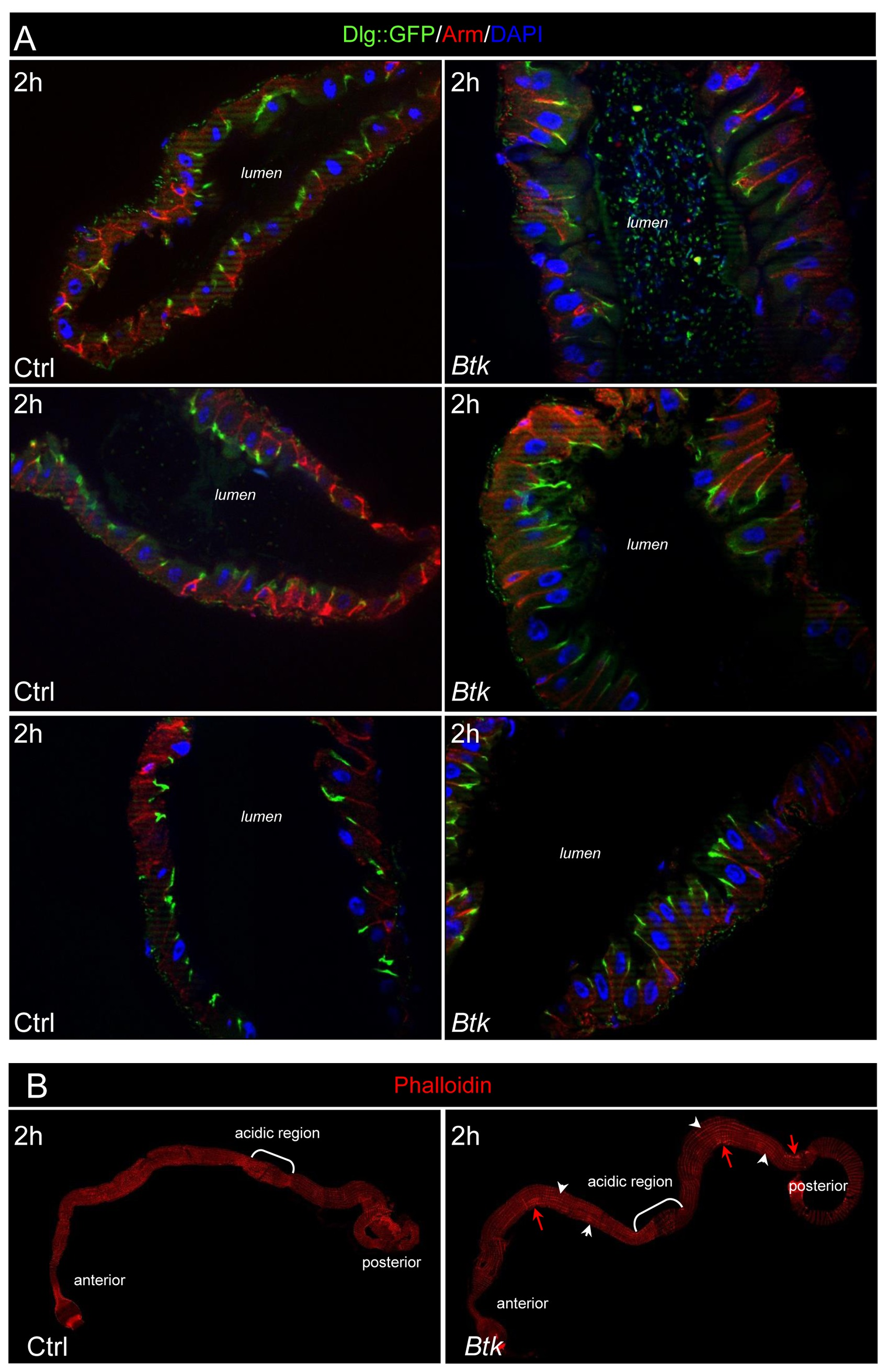

Supplement: S3 Fig — (A) Transversal cross-section of 3 independent posterior midguts 2h post ingestion of 5% sucrose (Ctrl) or Btk. DAPI labels the nuclei (Blue), Arm marks the basolateral compartment (Red) and Dlg::GFP marks the apical compartment. Objective is 40X. Note the elongated shapes of enterocytes in Btk-fed conditions. (B) Reconstructed image of midguts (10X objective) labelled with Phalloidin. 2h after ingestion of Btk some longitudinal muscle fibers are contracted. These contractions are scattered all along the midgut (white arrowheads in right panel). Note also that the midgut is bent in the zones of strong visceral contractions (red arrows). (TIF) [file ppat.1007279.s003.tif]

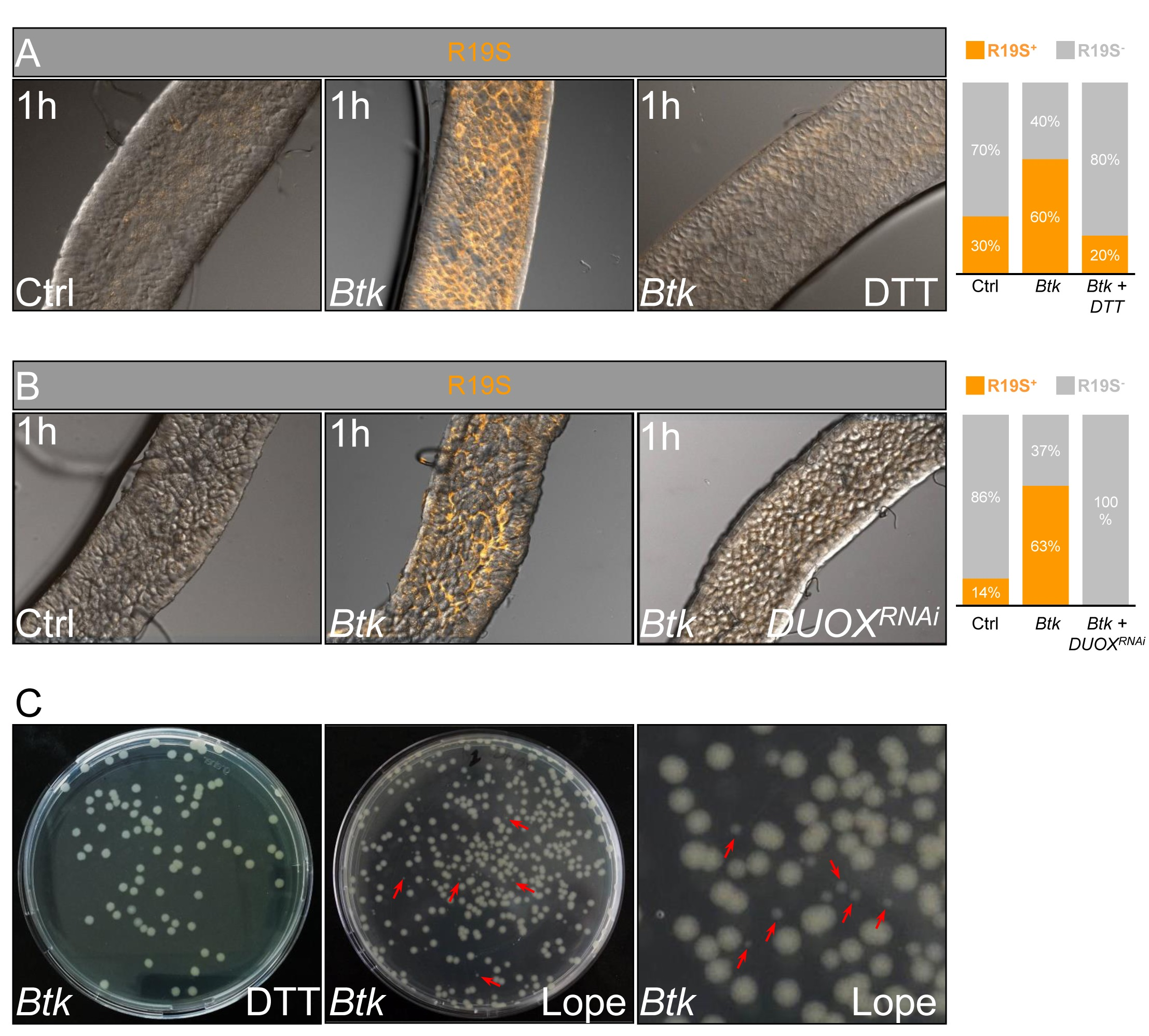

Supplement: S4 Fig — (A and B) Labelling of HOCl in anterior midgut by the R19S fluorescent probe (Orange) 1h after ingestion of sucrose 5% (Ctrl) or Btk (108 CFU were provided). Btk ingestion induces a production of ROS in the anterior part of the midgut (compared middle panels to left panels). Co-ingestion of DTT (A) or silencing DUOX expression in enterocytes (myo1Ats>DUOXRNAI) (B) neutralizes the production of ROS normally induced by Btk (right panels). Graphs on the right represent the proportion of R19S-positive midguts (orange). (C) Petri dishes plated with midgut lysates coming from flies co-fed with Btk (108 CFU) and DTT (left panel) or loperamide (middle and right panels). Note that the production ROS (loperamide condition) impairs bacterial growth as illustrated by the presence of small Btk colonies (red arrows). (TIF) [file ppat.1007279.s004.tif]

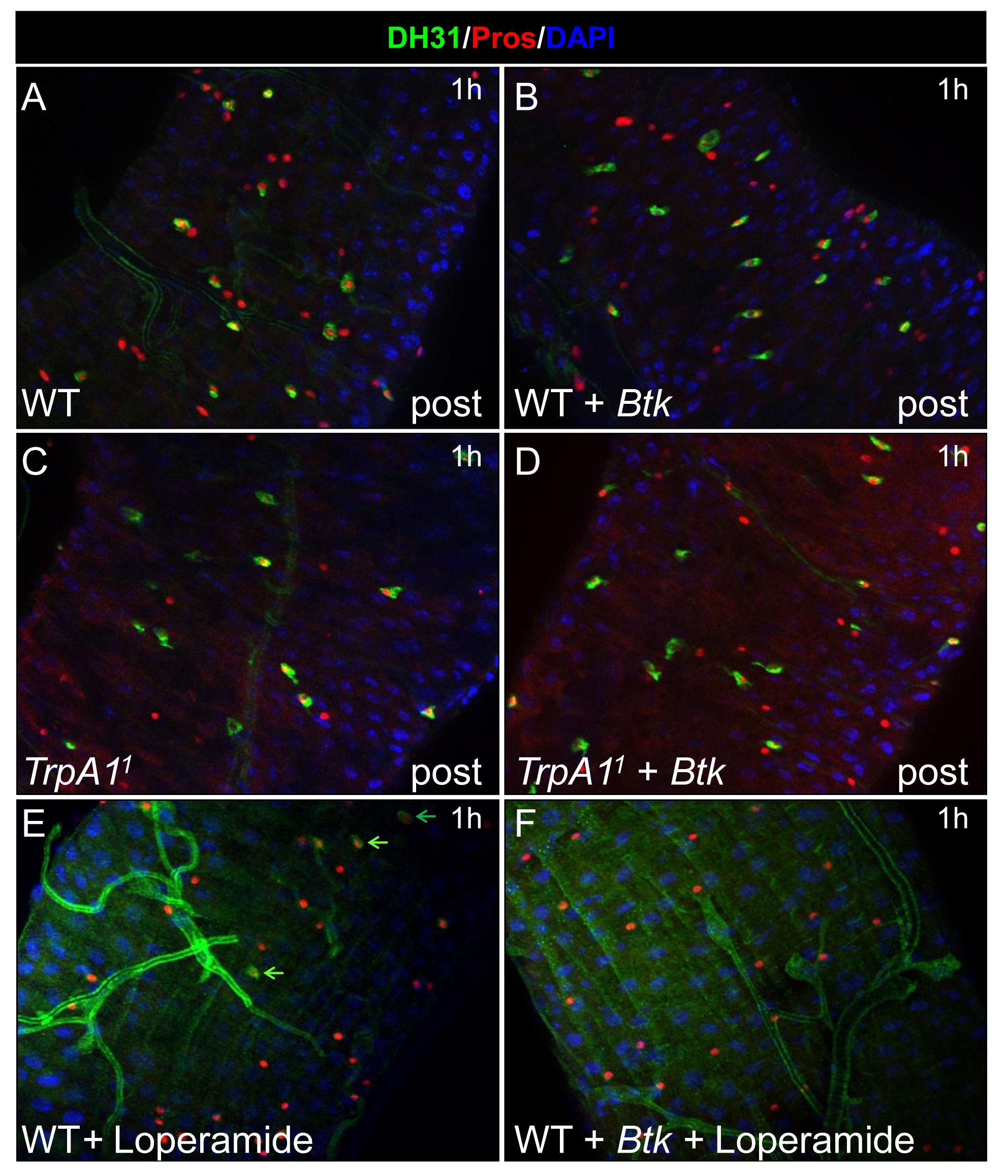

Supplement: S5 Fig — (A-F) Immunolabelling against DH31 (green) and Pros (red). DAPI (blue) marks the nuclei. (A and B) Posterior midguts of WT flies 1h post ingestion of sucrose (A) or 108 CFU of Btk (B). (C and D) Posterior midguts of TrpA11homozygote flies 1h post ingestion of sucrose (C) or 108 CFU of Btk (D). (E and F) Anterior midguts of WT flies 1h post ingestion of loperamide (E) or 108 CFU of Btk in presence of loperamide (F). (TIF) [file ppat.1007279.s005.tif]

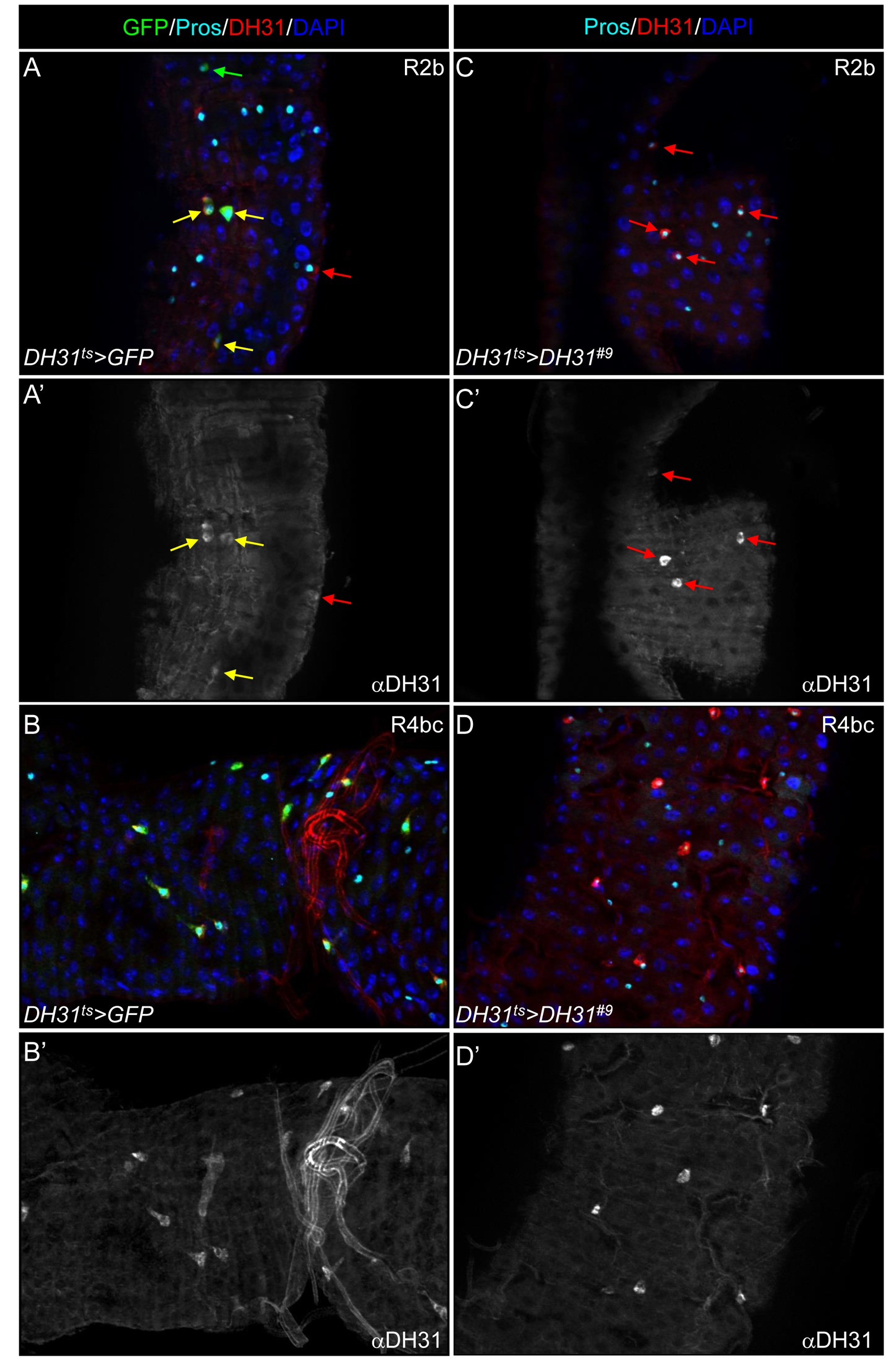

Supplement: S6 Fig — (A-D') Anti-Pros (turquoise) and anti-DH31 (red) immunostaining in DH31ts>GFP (A-B) or DH31ts>DH31 (C-D) midguts. Nuclei are marked with DAPI (blue). 40X objective. (A-A') R2b region in the anterior midgut. Note that in some GFP-expressing EECs, DH31 is below the threshold of detection (green arrow). There are also few EECs where DH31 is detectable without being marked by the GFP (red arrow) suggesting that the DH31-Gal4 driver (Bloomington stock #46389) does not perfectly recapitulate endogenous DH31 expression in the anterior midgut. (B-B') R4 region in the posterior midgut. GFP expression perfectly overlaps DH31-positive EECs. (C-C') Notable DH31 overexpression in the R2b region relative to the level of endogenous expression (compare C' to A'). (D-D') Overexpression of DH31 in the R4 region compared with (B-B'). (TIF) [file ppat.1007279.s006.tif]

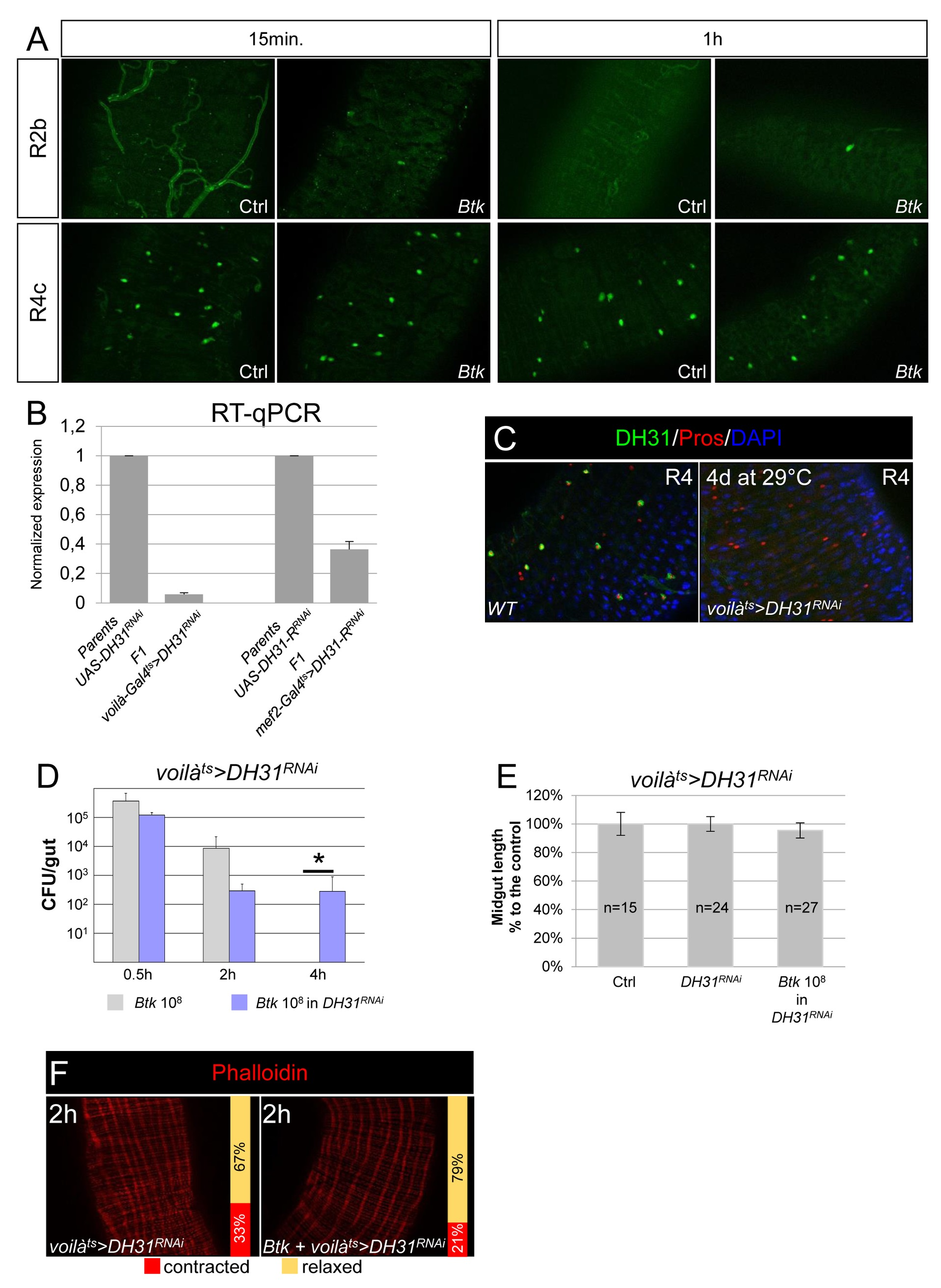

Supplement: S7 Fig — (A) Ca2+ release imaging (GFP) in DH31Gal4ts>GCaMP6s midguts. R2b and R4c regions were captured 15 min. and 1h post ingestion of Btk. Ctrl corresponds to flies fed with sucrose. (B) RT-qPCR on voilats>DH31RNAi or mef2ts>DH31-RRNAi whole midguts compared to RT-qPCR on their RNAi bearing parents. Normalized expression of DH31 (left) and DH31-R (right) are shown. (C) DH31 (green) and Pros (red) double immuno-labelling in WT (left) or voilats>DH31RNAi (right) posterior midgut. After 4 days of silencing, DH31 peptide is barely detectable in posterior EECs. Blue (DAPI) marks the nuclei. 40X objective. (D) CFU counting in the midgut of control (grey bars) or voilats>DH31RNAi flies (blue bars) fed with 108 CFU of Btk. (E) Measure of midgut length 2h post-intoxication of voilats>DH31RNAi flies fed or not with 108 CFU of Btk and compared to midgut length of control flies fed with 5% sucrose. (F) Phalloidin staining of posterior midgut of voilats>DH31RNAi flies 2h after feeding with 5% sucrose (left) or 108 CFU of Btk (right). 20X objective. (TIF) [file ppat.1007279.s007.tif]

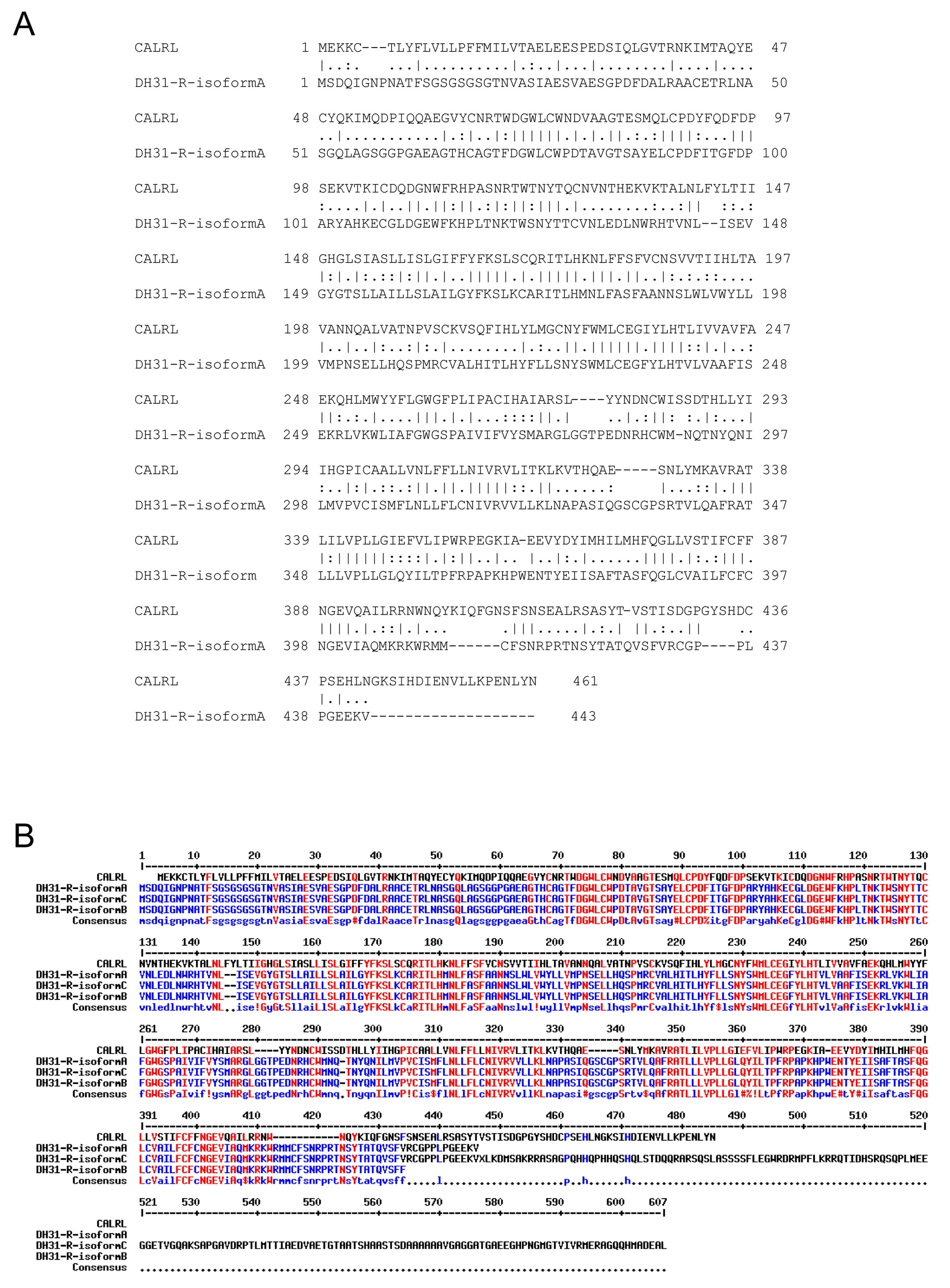

Supplement: S8 Fig — (A) The DH31-R isoform A amino acids sequence was aligned with human CALCRL amino acids sequence using needle program (http://emboss.toulouse.inra.fr/) with the following parameters: Matrix: EBLOSUM62; Gap penalty: 15.0; Extend penalty: 0.5. The two coding sequences present 34.5% of identity and 48.4% of similarity. (B) Alignment of the three DH31-R isoforms with human CALCRL (http://multalin.toulouse.inra.fr/multalin/). In red are shown the conserved amino acids. (TIF) [file ppat.1007279.s008.tif]
